# Consumption of Meals Prepared at Home and Risk of Type 2 Diabetes: An Analysis of Two Prospective Cohort Studies

**DOI:** 10.1371/journal.pmed.1002052

**Published:** 2016-07-05

**Authors:** Geng Zong, David M. Eisenberg, Frank B. Hu, Qi Sun

**Affiliations:** 1 Department of Nutrition, Harvard T.H. Chan School of Public Health, Boston, Massachusetts, United States of America; 2 Department of Epidemiology, Harvard T.H. Chan School of Public Health, Boston, Massachusetts, United States of America; 3 Channing Division of Network Medicine, Department of Medicine, Brigham and Women’s Hospital and Harvard Medical School, Boston, Massachusetts, United States of America; University of Cambridge, UNITED KINGDOM

## Abstract

**Background:**

There has been a trend towards increased dining out in many countries. Consuming food prepared out of the home has been linked to poor diet quality, weight gain, and diabetes risk, but whether having meals prepared at home (MPAH) is associated with risk of type 2 diabetes (T2D) remains unknown.

**Methods and Findings:**

We followed 58,051 women (from 1986 to 2012) and 41,676 men (from 1986 to 2010) in two prospective cohort studies of health professionals. Frequencies of consuming midday or evening MPAH were assessed at baseline and during follow-up. Incident T2D was identified through self-report and confirmed using a validated supplementary questionnaire. During 2.1 million person-years of follow-up, 9,356 T2D cases were documented. After adjusting for demographic, socioeconomic, and lifestyle factors, hazard ratios (HRs) and 95% confidence intervals (95% CIs) of T2D were 1 (reference), 0.93 (0.88–0.99), 0.96 (0.90–1.03), and 0.86 (0.81–0.91) for those eating 0–6, 7–8, 9–10, and 11–14 MPAH (*p*-trend < 0.001) per week, respectively. Participants eating 5–7 midday MPAH had 9% lower T2D risk than those with 0–2 midday MPAH, and participants having 5–7 evening MPAH had 15% lower risk than those with 0–2 evening MPAH (both *p*-trend < 0.001). In the first 8 y of follow-up, women and men who consumed 11–14 MPAH per week had 0.34 kg (95% CI: 0.15–0.53; *p* < 0.001) and 1.23 kg (95% CI: 0.92–1.54) less weight gain than those with 0–6 MPAH, respectively. Among participants who were nonobese (body mass index [BMI] < 30 kg/m^2^) at baseline, pooled HR (95% CI) of developing obesity (BMI ≥ 30 kg/m^2^) was 0.86 (0.82–0.91; *p*-trend < 0.001) when extreme MPAH groups (11–14 MPAH versus 0–6 MPAH) were compared. When midday and evening MPAH were analyzed separately, HRs comparing extreme groups (5–7 MPAH versus 0–2 MPAH) were 0.93 (95% CI: 0.89–0.97, *p*-trend = 0.003) for midday MPAH and 0.76 (95% CI: 0.70–0.83; *p*-trend < 0.001) for evening MPAH. Further adjusting for BMI during follow-up attenuated the association between MPAH and T2D risk: the HR (95% CI) for participants with 11–14 MPAH was 0.95 (0.89–1.01, *p*-trend = 0.13). The main limitations of our study were that it lacked assessments on individual foods constituting the MPAH and that the findings were limited to health professionals with a relatively homogeneous socioeconomic status.

**Conclusions:**

In two large prospective cohort studies, frequent consumption of MPAH is associated with a lower risk of developing T2D, and this association is partly attributable to less weight gain linked with this dining behavior.

## Abstract 2

With the aim of informing healthy eating behaviors, Qi Sun and colleagues investigate associations between the proportion of meals consumed by US health professionals that were prepared at home and incidence of type 2 diabetes.

## Author Summary

**Why Was This Study Done?:**

- Dining out has increasingly played an important role in daily diet in the United States and many other countries.
- Higher consumption of meals prepared out of home (MPOH), especially fast food, has been linked to lower dietary quality, excess weight gain, and an elevated risk of type 2 diabetes (T2D).
- Whether consuming meals prepared at home (MPAH) is associated with T2D risk remains unknown.

**What Did the Researchers Do and Find?:**

- We analyzed data for 58,051 US women followed from 1986 to 2012 and 41,676 US men followed from 1986 to 2010, who reported the frequencies of having midday or evening MPAH per week at baseline and during follow-up.
- Having MPAH more often was associated with a lower T2D risk in both men and women: those eating 11–14 MPAH per week had a 14% (95% confidence interval: 9%–19%) lower risk of developing T2D than those eating 0–6 MPAH per week.
- Men and women eating 11–14 MPAH per week had less weight gain compared to those eating 0–6 MPAH per week.
- The association between MPAH frequency and T2D risk could be partially ascribed to less body weight gain related to consumption of MPAH.
- The associations with obesity and T2D were stronger for evening MPAH than for midday MPAH.

**What Do These Findings Mean?:**

- Our findings suggest that people who eat MPAH more frequently have a lower long-term risk of developing T2D, and that this association is partially explained by less weight gain over time.
- Taken together with evidence from previous studies that focused on MPOH, these findings suggest that eating more MPAH instead of MPOH (especially fast foods) may help curb the risk of developing obesity and diabetes.
- From a public health perspective, actions are needed to encourage cooking meals at home and to improve diet quality of MPOH to facilitate diabetes prevention.

## Introduction

Dining out has become increasingly popular in many countries [1–5]. In the US, percentages of calorie intake attributed to meals prepared out of home (MPOH) increased from <10% in 1965–1966 to >31% in 2005–2008, and this has been accompanied by a significantly reduced time spent on cooking [2,3,6]. According to recent national survey data, 35%, 28%, and 61% of American adults ate in fast-food restaurants, full-service restaurants, and nonrestaurant sources of MPOH daily [7], with more than 10% of their energy intake coming from fast foods regardless of income levels [8].

MPOH are usually high in energy and fat but low in micronutrients, such as calcium, vitamin C, and iron [9]. One study reported that participants consuming MPOH had lower serum concentrations of vitamins D, E, C, B-6, B-12, folate, and carotenoids [10]. This less-optimal nutritional profile has raised public health concerns regarding the impact of eating out on long-term well-being, especially in relation to obesity and related chronic diseases [2,11]. In support of this, existing studies have reported a positive association between MPOH, especially fast-food intake, and weight gain among children and young adults [12], although data for diabetes are relatively sparse [13–16].

More frequent consumption of MPOH is often at the cost of consuming fewer meals prepared at home (MPAH) [2,17]. In contrast to accumulating evidence regarding potential adverse effects of MPOH or fast foods, few studies have explicitly evaluated consuming MPAH in relation to health consequences. Previously, eating dinner cooked at home has been associated with lower intakes of fat and sugar [17], but whether eating MPAH is associated with obesity and obesity-related diseases remains largely unknown. To narrow the knowledge gap, we investigated the association between frequencies of consuming midday and evening MPAH and the risk of type 2 diabetes (T2D) in middle-aged and older US men and women.

## Methods

### Ethics Statement

The study protocol was approved by the institutional review boards of the Brigham and Women's Hospital and the Harvard T. H. Chan School of Public Health. Completion of the self-administered questionnaire was considered to imply informed consent.

### Study Populations

The current investigation was based on two prospective cohort studies: the Nurses’ Health Study (NHS), which includes 121,700 female registered nurses aged 30–55 y who were enrolled in 1976 [18], and the Health Professionals Follow-up Study (HPFS), which consists of 51,529 male health professionals aged 40–75 y who were enrolled in 1986 [19]. Participants in both studies have been followed through mailed biennial questionnaires to collect and update information on lifestyles, health-related behaviors, and medical histories. Active follow-up rates exceed 90% in both cohorts.

In the current investigation, the study baseline was 1986 when information on MPAH was collected in both cohorts. Among the 75,521 participants in NHS and 51,529 in HPFS with completed baseline food frequency questionnaire (FFQ), we excluded those who reported a diagnosis of diabetes, cardiovascular diseases, or cancer at baseline (*n* = 10,828 in NHS, and *n* = 6,933 in HPFS); had daily energy intake out of a normal range (<500 or >3,500 kcal/d for NHS and <800 or >4,200 kcal/d for HPFS) or missing data of baseline MPAH (*n* = 5,889 in NHS, and *n* = 1,507 in HPFS); had a missing diagnosis date of T2D (*n* = 262 in HPFS); completed the baseline questionnaire only or with missing age at baseline (*n* = 753 in NHS, and *n* = 1,151 in HPFS), leaving 58,051 women and 41,676 men for the analyses (S1 Fig). In both NHS and HPFS, the distribution of demographic, socioeconomic, anthropometric, and lifestyle factors were similar between participants who had missing MPAH data and those included in the final analysis.

### Ascertainment of Diet and Frequencies of MPAH

Since 1986 and every 4 y thereafter, a validated 138-item FFQ was sent to participants in both cohorts to assess and update their habitual diet during the past year [20]. We calculated cumulative averages of diet based on valid assessments from baseline to the end of follow-up and stopped updating dietary information if participants reported a diagnosis of diabetes, cardiovascular disease, or cancer [20]. Nutrient intakes were adjusted for total energy intake using the residual method [21]. The 2010 Alternative Health Eating Index (AHEI) was calculated as described previously with alcohol consumption excluded [20].

In 1986, participants were asked the following two questions: “How often your midday meals were prepared at home” and “How often your evening meals were prepared at home,” with five prespecified response categories in the NHS (never, 1–2, 3–4, 5–6, and 7 times/week) and four categories in HPFS (never, 1–2, 3–4, and 5–7 times/week). We summed midpoint values of midday and evening MPAH in each category to generate the overall MPAH frequency. To ensure an adequate number of T2D cases in each comparison group while maintaining a meaningful variation, we categorized overall MPAH frequency into four groups (0–6, 7–8, 9–10, or 11–14 times/week) and frequency of consuming midday or evening MPAH into three groups (0–2, 3–4, or 5–7/week). In the 2002 questionnaire of NHS and the 2004 and 2006 questionnaires of HPFS, the frequency of eating MPAH (either lunch or dinner) was assessed again, and response categories were changed to the following: almost none, 25%, 50%, 75%, and almost all. For the analysis of overall MPAH, we matched the same categories to the 2002–2006 MPAH question as follows: “almost none” and “25%” were assigned to 0–6 MPAH, “50%” to 7–8 MPAH, “75%” to 9–10 MPAH, and “almost all” to 11–14 MPAH. We used this updated MPAH frequency to account for potential changes in diet behavior and food environment during the long follow-up. The analysis for midday and evening MPAH used baseline MPAH frequency only.

### Ascertainment of Incident T2D

In both cohorts, participants who reported a diagnosis of diabetes were mailed a supplementary questionnaire regarding symptoms, diagnostic tests, and hypoglycemic therapy. The diagnosis of T2D was considered confirmed if at least one of the following was reported on the supplementary questionnaire according to the National Diabetes Data Group criteria [22]: (1) one or more classic symptoms (excessive thirst, polyuria or frequent urination, weight loss, and hunger) plus fasting plasma glucose ≥7.8 mmol/l or random plasma glucose levels ≥11.1 mmol/l; (2) ≥2 elevated plasma glucose concentrations on different occasions (fasting glucose ≥7.8 mmol/l, random plasma glucose ≥11.1 mmol/l, and/or plasma glucose ≥11.1 mmol/l after ≥2 h shown by oral glucose tolerance testing) in the absence of symptoms; or (3) treatment with hypoglycemic medication (insulin or oral hypoglycemic agent). The diagnostic criteria changed in June 1998, and a fasting plasma glucose of 7.0 mmol/l was considered the threshold for the diagnosis of diabetes instead of 7.8 mmol/l according to the American Diabetes Association criteria [23]. In validation studies, 61 of 62 self-reported cases of T2D in the NHS who were confirmed by the supplementary questionnaire were reconfirmed after an endocrinologist reviewed the medical records [24]. In the HPFS, 57 of 59 self-reported diabetes cases (97%) were reconfirmed by medical record review [25].

### Assessment of Covariables

Information on anthropometric, demographic, socioeconomic, and lifestyle factors, including body weight, marital status, employment status, number of children, cigarette smoking, physical activity, family history of diabetes, multivitamin use, menopausal status, and hormone use, was updated in follow-up questionnaires. Physical activity was estimated by multiplying the energy expenditure in metabolic equivalent tasks (METs) measured in hours per week by hours spent on the activity, and the values of all activities were summed to derive total physical activity. Body mass index (BMI) was calculated as self-reported weight in kilograms divided by height in meters squared.

### Statistical Analysis

To minimize sample reduction because of missing covariables, we used indicator variables for missing categorical variables (cigarette smoking, marital status, employment status, number of children, menopausal status, and postmenopausal hormone use) at baseline and follow-up. The overall percentages of missing values during follow-up ranged from 0.5% (for cigarette smoking) to 7.4% (for employment status) in the NHS, and from 0.1% (for physical activity) to 23.0% (for employment status) in the HPFS. For BMI and physical activity, missing data during follow-up were replaced with the last valid values, and dummy variables were created for missing baseline data when making categories. The overall percentages of missing data for BMI and physical activity were 0.3% and 6.1% in the NHS and 0.3% and 0.1% in the HPFS, respectively. Excluding employment status as a covariable or restricting analysis among participants with valid data of employment status did not change the results.

We calculated Spearman’s rank correlation coefficients between midday and evening MPAH and the correlation coefficients of MPAH frequency assessed at baseline and during follow-up. Person-years were calculated from the return of baseline FFQ to the diagnosis of T2D, last return of a valid follow-up questionnaire, death, or end of follow-up (2012 in NHS and 2010 in HPFS), whichever came first. Cox proportional hazards models were used to calculate hazard ratios (HRs) and 95% confidence intervals (95% CIs) for the association between frequencies of consuming MPAH and T2D risk. To account for confounding of age and time, the Cox regression analysis was stratified by age in months and calendar year. Multivariable models were adjusted for ethnicity (Caucasian, African American, Hispanic, or Asian), marital status (married, not married, or missing), employment status (full-time, part-time, retired, or missing), number of children (none, 1–2, 3–4, 5 or more, or missing), smoking status (never smoked, past smoker, currently smokes 1–14 cigarettes/d, currently smokes 15–24 cigarettes/d, currently smokes ≥25 cigarettes/d, or missing), alcohol intake (gram/d: 0, 0.1–4.9, 5.0–14.9, or >15.0 in women; 0, 0.1–4.9, 5.0–29.9, or >30.0 in men; or missing), multivitamin use (yes or no), family history of diabetes (yes or no), menopause status and postmenopausal hormones use (women only: premenopause, never, former, or current hormone use, or missing), physical activity (METs/week: 0–2.9, 3–8.9, 9–17.9, 18–26.9, ≥27.0, or missing), and total energy intake (kcal/d). We adjusted for employment status, number of children, and marital status in both cohorts to control for potential confounding by socioeconomic status. In a secondary analysis of NHS data, we further adjusted for the highest degree acquired (registered nurse, bachelor’s degree, master’s degree and above, or missing), the education attainment of participant’s husband (some high school or below, high school graduate, college graduate, graduate school, or missing), participant’s perception on her standing in US society (top 20%, 30%, 40%, 50%, >50%, or missing), and participant’s perception on her standing in local community (top 20%, 30%, 40%, 50%, >50%, or missing) to explore whether the associations of interest are independent of these socioeconomic variables. We mutually adjusted for midday MPAH and evening MPAH when modeling their associations with T2D to control for potential confounding by each other. The linear trend was tested by modeling the median values for frequencies of MPAH categories as a continuous variable. HRs from the two cohorts were pooled using a fixed effects model, and the Cochrane Q statistic and the *I* ^2^ statistic were used to examine the heterogeneity of associations between the two cohorts.

We examined the association between MPAH and weight gain from baseline to 1994 when the mean age of the study participants reached 60 y [26], because differential body weight loss at an older age may obscure true associations of interest [27]. Differences in weight changes across the MPAH groups were estimated using a general linear model, after excluding participants with missing body weight at baseline or with only baseline weight available till 1994 (*n* = 4,930 in NHS, and *n* = 2,436 in HPFS). Among nonobese participants (defined by BMI < 30 kg/m^2^) at baseline (*n* = 49,355 in NHS, and *n* = 38,511 in HPFS), we additionally estimated the risk of developing obesity. Multivariable models were adjusted for the same covariables considered in the T2D analysis and further adjusted for baseline BMI for weight change.

Based on existing evidence suggesting that the frequency of eating out may influence food choices and weight change, which are also risk factors for T2D [9,12], we further evaluated whether BMI, overall dietary quality (as reflected by AHEI), and individual dietary factors (carbonated beverages, sodium, total fruits, total vegetables, red meats, processed meats, total dairy products, coffee, French fries, whole grains, the polyunsaturated fatty acids to saturated fatty acids ratio, and trans fat; in quintiles) during follow-up may explain the associations between MPAH and T2D. We estimated the proportion of associations between MPAH and diabetes that was attributable to these factors using a SAS macro developed by Spiegelman et al. (http://www.hsph.harvard.edu/donna-spiegelman/software/mediate/) [28]. We also adjusted for frequencies of consuming fried food away from home to explore whether the association between MPAH and T2D is independent of the energy-dense, obesogenic MPOH.

To test the robustness of associations between MPAH and T2D, we censored participants when they retired or divorced/separated from their partner after baseline survey, because these changes may possibly alter cooking frequency and dining behaviors, thus introducing extraneous variability into the associations of interest. To further exclude the possibility that participants with a very high risk of T2D or prediabetes may reduce their frequency of eating out in an effort to pursue a healthier diet, we excluded participants who reported incident T2D in the first 4 y of follow-up. We also conducted stratified analyses by age (<65 y, ≥65 y), BMI (<30 kg/m^2^, ≥30 kg/m^2^), physical activity (<18 METs/week, ≥18 METs/week), and smoking status (currently smoking or not) to determine potential modifying effects of these factors. Time-varying variables were used in stratified analyses, and the *p*-values for the product terms between median frequency of MAPH categories and stratification variables were used to evaluate the significance of interactions.

All statistical analyses were conducted using SAS 9.4 (SAS Institute, Cary, North Carolina), and *p*-values were two-sided with a significance level of 0.05.

## Results

The baseline characteristics of the study participants are shown in Table 1. The mean age of the study populations was 52.5 y (women: 52.0 y; men: 53.0 y). In both cohorts, those reporting more frequent consumption of MPAH were older, with more children, and more likely to be married, retired, and nonsmokers. Physical activity was higher in women eating more MPAH, but the opposite was observed in men. Regarding dietary factors, men and women eating more MPAH had higher intakes of total calories, fruits, vegetables, red meats, dairy products, and whole grains and lower intakes of carbonated beverages and coffee. Women with different MPAH frequencies had similar intake of fried foods prepared out of home or at home, whereas men eating more MPAH had lower consumption of fried food cooked away from home and higher intake of fried food prepared at home. The distribution of participants’ characteristics was similar when the study populations were categorized according to either midday or evening MPAH (S1 and S2 Tables).

**Table 1:** Baseline characteristics in the NHS and the HPFS (1986) according to frequencies of consuming MPAH.

Variables^a^	Frequency of Consuming MPAH, Times/Week							
	NHS				HPFS			
	0–6	7–8	9–10	11–14	0–6	7–8	9–10	11–14
Number of participants	10,551	18,273	8,553	20,674	12,366	13,295	5,538	10,477
Number of midday meals prepared at home	1.1 (0.5)	1.4 (1.1)	4.0 (0.9)	6.0 (0)	1.0 (0.3)	1.4 (1.0)	3.9 (1.0)	6.0 (0)
Number of evening meals prepared at home	3.9 (1.7)	5.6 (1.1)	5.5 (0.9)	6.0 (0)	3.7 (1.6)	5.6 (1.0)	5.6 (1.0)	6.0 (0)
Age, years	50.8 (6.6)	50.7 (6.6)	51.7 (7.2)	54.1 (7.3)	51.0 (8.6)	51.5 (8.9)	54.2 (9.9)	56.7 (9.9)
Race, white, %	97	97	98	99	94	95	95	96
Married, %	73	81	86	88	84	93	93	95
Number of children	2.7 (1.6)	2.8 (1.6)	2.9 (1.6)	3.0 (1.8)	2.6 (1.6)	2.8 (1.6)	2.8 (1.6)	2.9 (1.7)
Currently working, %	81	81	70	54	94	93	88	84
Family history of diabetes, %	25	26	25	26	19	19	19	19
Physical activity, METs/week	13.5 (20.5)	13.0 (18.0)	14.9 (19.9)	15.2 (21.6)	22.6 (31.0)	20.7 (28.5)	21.5 (29.6)	20.5 (29.5)
Currently smoking, %	28	22	18	18	10	9	9	9
Alcohol intake, g/day	6.8(11.4)	6.1(10.3)	6.5(10.5)	6.0(10.5)	11.5(15.4)	11.2(14.8)	11.3(15.2)	11.3(16.0)
Multivitamin use,%	43	41	43	43	43	41	42	40
Any use of postmenopausal hormone, %	27	26	27	26	-	-	-	-
BMI, kg/m^2^	25.1(4.7)	25.3(4.7)	25.1(4.5)	24.9(4.6)	25.0(5.1)	24.9(4.9)	25.0(4.7)	24.8(4.7)
Dietary variables								
Total energy, kcal/day	1,638 (529)	1,748 (519)	1,822 (527)	1,845 (517)	1,899 (625)	1,987 (614)	2,040 (620)	2,099 (610)
Total fruits, servings/day	2.2 (1.5)	2.5 (1.5)	2.6 (1.5)	2.7 (1.5)	2.3 (1.7)	2.3 (1.5)	2.4 (1.6)	2.5 (1.6)
Total vegetables, servings/day	2.8 (1.6)	3.1 (1.6)	3.2 (1.6)	3.3 (1.6)	2.9 (1.7)	3.1 (1.6)	3.1 (1.7)	3.1 (1.7)
Red meats, servings/day	0.9 (0.6)	1.1 (0.6)	1.1 (0.7)	1.1 (0.7)	1.0 (0.8)	1.2 (0.8)	1.2 (0.8)	1.3 (0.9)
Processed meats, servings/day	0.1 (0.2)	0.1 (0.2)	0.1 (0.2)	0.1 (0.2)	0.2 (0.3)	0.2 (0.3)	0.2 (0.2)	0.2 (0.3)
Total dairy products, servings/day	2.0 (1.4)	2.1 (1.4)	2.2 (1.3)	2.3 (1.4)	1.7 (1.3)	1.9 (1.4)	2.0 (1.4)	2.2 (1.5)
Carbonated beverages, servings/day	0.9 (1.2)	0.8 (1.1)	0.8 (1.0)	0.7 (1.0)	0.9 (1.1)	0.8 (1.0)	0.7 (0.9)	0.6 (0.9)
Coffee consumption, cups/day	2.7 (1.9)	2.5 (1.8)	2.3 (1.7)	2.3 (1.7)	4.4 (2.9)	4.3 (2.9)	4.1 (2.9)	3.8 (3.0)
French fries, servings/day	0.1 (0.1)	0.1 (0.1)	0.1 (0.1)	0.1 (0.1)	0.1 (0.2)	0.1 (0.1)	0.1 (0.1)	0.1 (0.1)
Whole grains, g/day	13.2 (13.9)	13.6 (13.4)	14.3 (13.0)	15.3 (14.1)	20.0 (19.6)	21.4 (18.7)	22.8 (19.2)	23.4 (19.9)
Trans fatty acids, % energy	1.6 (0.5)	1.7 (0.5)	1.7 (0.5)	1.7 (0.5)	1.3 (0.5)	1.3 (0.5)	1.3 (0.5)	1.3 (0.5)
P/S ratio	0.5 (0.2)	0.5 (0.2)	0.6 (0.2)	0.6 (0.2)	0.6 (0.2)	0.6 (0.2)	0.6 (0.2)	0.6 (0.2)
Sodium, g/day	2.8 (1.1)	2.9 (1.0)	2.8 (1.0)	2.8 (1.0)	3.2 (1.2)	3.3 (1.1)	3.3 (1.1)	3.3 (1.1)
AHEI^b^	45.3 (10.3)	45.4 (10.2)	45.7 (10.1)	45.9 (10.6)	47.0 (10.7)	46.5 (10.6)	46.8 (10.8)	46.2 (11.1)
Frequency of eating fried food away from home^c^	0.6 (0.4)	0.6 (0.3)	0.6 (0.3)	0.5 (0.3)	1.6 (1.4)	1.3 (1.2)	1.3 (1.0)	0.9 (0.7)
Frequency of eating fried food at home^c^	1.1 (0.8)	1.2 (0.9)	1.2 (0.9)	1.2 (0.9)	1.2 (1.1)	1.4 (1.5)	1.5 (1.3)	1.7 (1.5)

Abbreviations: AHEI, Alternative Health Eating Index; BMI, body mass index; HPFS, Health Professionals Follow-up Study; MPAH, meals prepared at home; NHS, Nurses’ Health Study, MET, metabolic equivalent tasks; P/S ratio, polyunsaturated fatty acids and saturated fatty acids ratio.

^a^ Values are means (standard deviations) or percentages standardized to the age distribution of the study population.

^b^ Alcohol consumption was not included in the AHEI score.

^c^ 'Frequency' refers to number of times per day.

Spearman’s rank correlation coefficients between frequencies of midday and evening MPAH were 0.19 in women and 0.26 in men (*p* < 0.001 for both). The correlations of MPAH frequencies at baseline and during follow-up were 0.22 (*p* < 0.001) between the 1986 and 2002 assessments for NHS, 0.34 (*p* < 0.001) between the 1986 and 2004 assessments, or 0.33 (*p* < 0.001) between the 1986 and 2006 assessments in HPFS. The correlation coefficient between the 2004 and 2006 MPAH assessments in HPFS was 0.69 (*p* < 0.001), suggesting that the frequency of eating MPAH was stable within a shorter period of time.

During 2.1 million person-years of follow-up (mean follow-up duration was 21 y), 9,356 cases of incident diabetes were documented. Associations between the frequency of consuming MPAH and T2D risk are shown in Table 2. In the age-adjusted model, increased consumption of MPAH was significantly associated with a lower risk of T2D. Findings did not materially change after further adjustment for other demographic and socioeconomic factors (model 2) or lifestyle factors (model 3). In model 3, pooled HRs (95% CIs) of T2D were 1 (reference), 0.93 (0.88–0.99), 0.96 (0.90–1.03), and 0.86 (0.81–0.91) for participants consuming 0–6, 7–8, 9–10, and 11–14 MPAH per week (*p*-trend < 0.001), respectively. As shown in S3 Table, the association between overall MPAH and diabetes risk remained when only baseline MPAH assessments were used for the analysis. Both midday and evening MPAH frequencies were associated with a lower risk of T2D, before or after adjustment for confounding factors. When midday and evening MPAH were mutually adjusted (model 4), participants eating 5–7 midday MPAH had a 9% (95% CI: 4%–13%; *p*-trend < 0.001) lower risk of T2D compared to the 0–2 midday MPAH group. For evening meals, people with 5–7 MPAH had a 15% (95% CI: 6%–22%; *p*-trend < 0.001) lower risk of T2D than those with 0–2 MPAH.

**Table 2:** Hazard ratios (95% confidence intervals) of type 2 diabetes according to frequencies of meals prepared at home in the Nurses’ Health Study (1986–2010) and the Health Professionals Follow-up Study (1986–2010).

	Frequencies of Consuming MPAH, Times/Week				*P*-trend
Overall MPAH	0–6	7–8	9–10	11–14	
NHS					
Cases/person-years	898/191,094	1,556/334,966	1,272/254,374	2,228/501,428	
Model 1^a^	1.00	0.99 (0.91–1.07)	0.95 (0.87–1.04)	0.87 (0.80–0.94)	<0.001
Model 2^b^	1.00	0.99 (0.91–1.07)	0.98 (0.90–1.07)	0.88 (0.81–0.96)	0.001
Model 3^c^	1.00	0.97 (0.90–1.06)	0.99 (0.91–1.09)	0.86 (0.80–0.94)	<0.001
HPFS					
Cases/person-years	994/230,694	964/255,276	537/130,864	916/232,619	
Model 1^a^	1.00	0.87 (0.80–0.96)	0.92 (0.83–1.03)	0.87 (0.79–0.95)	0.005
Model 2^b^	1.00	0.88 (0.80–0.96)	0.90 (0.81–1.00)	0.85 (0.77–0.93)	0.001
Model 3^c^	1.00	0.88 (0.81–0.97)	0.92 (0.83–1.03)	0.85 (0.77–0.94)	0.002
Pooled^e^					
Model 2^b^	1.00	0.93 (0.88–0.99)	0.95 (0.88–1.02)	0.87 (0.81–0.92)	<0.001
*P*-heterogeneity	−	0.05	0.23	0.54	0.69
Model 3^c^	1.00	0.93 (0.88–0.99)	0.96 (0.90–1.03)	0.86 (0.81–0.91)	<0.001
*P*-heterogeneity	–	0.12	0.30	0.86	0.95
Midday MPAH	0–2	3–4	5–7		
NHS					
Cases/person-years	2,817/590,330	933/200,313	2,204/491,219		
Model 1^a^	1.00	0.98 (0.91–1.06)	0.94 (0.88–0.99)		0.02
Model 2^b^	1.00	1.00 (0.93–1.08)	0.95 (0.89–1.01)		0.09
Model 3^c^	1.00	1.00 (0.92–1.07)	0.91 (0.85–0.96)		0.002
Model 4^d^	1.00	1.00 (0.93–1.08)	0.92 (0.87–0.98)		0.01
HPFS					
Cases/person-years	2,049/492,274	499/129,942	863/227,237		
Model 1^a^	1.00	0.91 (0.83–1.01)	0.88 (0.81–0.96)		0.002
Model 2^b^	1.00	0.91 (0.83–1.01)	0.88 (0.81–0.96)		0.002
Model 3^c^	1.00	0.91 (0.83–1.01)	0.87 (0.80–0.95)		0.001
Model 4^d^	1.00	0.93 (0.84–1.02)	0.89 (0.81–0.97)		0.006
Pooled^e^					
Model 2^b^	1.00	0.97 (0.91–1.03)	0.93 (0.88–0.97)		0.001
*P*-heterogeneity	–	0.15	0.15		0.12
Model 3^c^	1.00	0.97 (0.91–1.03)	0.90 (0.85–0.94)		<0.001
*P*-heterogeneity	–	0.20	0.46		0.41
Model 4^d^	1.00	0.97 (0.92–1.03)	0.91 (0.87–0.96)		<0.001
*P*-heterogeneity	*–*	0.23	0.48		0.43
Evening MPAH	0–2	3–4	5–7		
NHS					
Cases/person-years	290/49,691	932/185,621	4,732/1,046,551		
Model 1^a^	1.00	0.87 (0.76–0.99)	0.78 (0.69–0.88)		<0.001
Model 2^b^	1.00	0.90 (0.79–1.03)	0.81 (0.72–0.91)		<0.001
Model 3^c^	1.00	0.95 (0.83–1.09)	0.83 (0.74–0.94)		<0.001
Model 4^d^	1.00	0.95 (0.83–1.08)	0.84 (0.75–0.95)		<0.001
HPFS					
Cases/person-years	208/42,450	853/205,134	2,350/601,870		
Model 1^a^	1.00	0.85 (0.73–0.99)	0.79 (0.69–0.92)		0.001
Model 2^b^	1.00	0.85 (0.74–0.99)	0.80 (0.69–0.92)		0.003
Model 3^c^	1.00	0.89 (0.76–1.04)	0.84 (0.73–0.97)		0.008
Model 4^d^	1.00	0.90 (0.78–1.06)	0.87 (0.75–1.01)		0.06
Pooled^e^					
Model 2^b^	1.00	0.88 (0.79–0.97)	0.81 (0.73–0.88)		<0.001
*P*-heterogeneity	–	0.57	0.86		0.72
Model 3^c^	1.00	0.93 (0.84–1.03)	0.84 (0.76–0.92)		<0.001
*P*-heterogeneity	–	0.58	0.94		0.45
Model 4^d^	1.00	0.93 (0.84–1.03)	0.85 (0.78–0.94)		<0.001
*P*-heterogeneity	–	0.73	0.73		0.31

^a^ Estimates are calculated in Cox proportional hazards models. Model 1, adjusted for age;

^b^ model 2 was further adjusted for ethnicity (Caucasian, African American, Hispanic, or Asian), marital status (married, not married, or missing), employment status (full-time work, part-time work, retirement, or missing), number of children (0, 1–2, 3–4, 5 or more, or missing), and family history of diabetes (yes or no) based on model 1;

^c^ model 3 was further adjusted for smoking status (never smoked, past smoker, or currently smokes 1–14 cigarettes/d, currently smokes 15–24 cigarettes/d, or currently smokes ≥25 cigarettes/d, or missing), alcohol intake (gram/d: 0, 0.1–4.9, 5.0–14.9, or >15.0 in women; 0, 0.1–4.9, 5.0–29.9, or >30.0 in men; or missing), multivitamin use (yes, no, or missing), menopause status and postmenopausal hormones use (women only: premenopause, postmenopause [never, former, or current hormone use], or missing), physical activity (METs/week: 0–2.9, 3–8.9, 9–17.9, 18–26.9, ≥27.0, or missing), and total energy intake (kcal/d) based on model 2;

^d^ model 4 was further mutually adjusted for midday or evening meals prepared at home based on model 3, i.e., the analysis of midday meals prepared at home was further adjusted for evening meals prepared at home, and vice versa;

^e^ study estimates from the two cohorts were pooled using a fixed-effects model.

During the follow-up period from 1986 to 1994, more frequent consumption of MPAH was associated with slower weight gain and a lower risk of developing obesity (Table 3). For women, significantly less weight gain (standard error) was observed only when extreme MPAH groups were compared (3.36 [0.06] kg for 0–6 MPAH versus 3.02 [0.05] kg for 11–14 MPAH; *p* < 0.001). For men, weight changes (standard error) were 3.85 (0.09, 0–6 MPAH) kg, 3.32 (0.08, 7–8 MPAH) kg, 3.34 (0.13, 9–10 MPAH) kg, and 2.62 (0.10, 11–14 MPAH) kg, and all groups with >6 MPAH had significantly less weight gain than the 0–6 MPAH group did (all *p* < 0.01 for between-group comparisons). Least squared mean differences in weight changes comparing the 11–14 with the 0–6 groups were −0.34 (95% CIs: −0.15 to −0.53; *p* < 0.001) kg in women and −1.23 (95% CIs: −0.92 to −1.54; *p* < 0.001) kg in men. Compared with the 0–6 MPAH group, pooled HR (95% CI) of developing obesity was 0.86 (0.82–0.91) for participants consuming 11–14 MPAH. Women with 5–7 midday MPAH had marginally less weight gain than those who ate 0–2 midday MPAH (*p*-trend = 0.06), whereas the same comparison reached significance in men (*p*-trend <0.01). For evening meals, men and women eating 5–7 evening MPAH gained less weight in comparison with the 0–2 evening MPAH groups (*p*-trend < 0.001). Consistently, participants eating 5–7 midday MPAH had a 7% lower risk of developing obesity (*p* < 0.001), whereas participants eating 5–7 evening MPAH had a 24% (*p* < 0.001) lower risk, compared with the corresponding 0–2 MPAH groups.

**Table 3:** Weight changes and risk of obesity according to frequencies of meals prepared at home, based on the NHS and the HPFS (1986–1994).

Variables^a^	Frequencies of Consuming MPAH, Times/Week				*P*-trend
Overall MPAH	0–6	7–8	9–10	11–14	
Weight Change, kg					
NHS	3.36 (0.06)	3.30 (0.05)	3.22 (0.07)	3.02 (0.05)^c^	<0.001
HPFS	3.85 (0.09)	3.32 (0.08)^c^	3.34 (0.13)^b^	2.62 (0.10)^c^	<0.001
Hazard Ratio of Obesity					
NHS	1.00	0.99 (0.92–1.05)	0.98 (0.92–1.05)	0.87 (0.82–0.93)	<0.001
HPFS	1.00	0.89 (0.80–0.99)	0.97 (0.84–1.12)	0.82 (0.72–0.93)	0.007
Pooled results^d^	1.00	0.97 (0.92–1.02)	0.98 (0.92–1.04)	0.86 (0.82–0.91)	<0.001
*P*-heterogeneity	–	0.10	0.89	0.40	0.60
Midday MPAH	0–2	3–4	5–7		
Weight Change, kg					
NHS	3.25 (0.04)	3.26 (0.07)	3.12 (0.05)		0.06
HPFS	3.52 (0.06)	3.46 (0.12)	2.79 (0.09)^c^		<0.001
Hazard Ratio of Obesity					
NHS	1.00	1.03 (0.97–1.09)	0.93 (0.89–0.98)		0.005
HPFS	1.00	1.09 (0.96–1.23)	0.93 (0.82–1.04)		0.34
Pooled results^d^	1.00	1.04 (0.98–1.09)	0.93 (0.89–0.97)		0.003
*P*-heterogeneity	–	0.44	0.92		0.87
Evening MPAH	0–2	3–4	5–7		
Weight Change, kg					
NHS	3.90 (0.14)	3.41 (0.07)^b^	3.13 (0.03)^c^		<0.001
HPFS	3.65 (0.21)	3.73 (0.10)	3.14 (0.06)^b^		<0.001
Hazard Ratio of Obesity					
NHS	1.00	0.82 (0.74–0.91)	0.76 (0.69–0.84)		<0.001
HPFS	1.00	0.85 (0.70–1.03)	0.77 (0.63–0.93)		0.003
Pooled results^d^	1.00	0.83 (0.75–0.90)	0.76 (0.70–0.83)		<0.001
*P*-heterogeneity	–	0.76	0.97		0.82

^a^ Estimates are least squared means (standard errors) from general linear model and hazard ratios (95% confidence intervals) from Cox proportional hazards models after adjusting for age, ethnicity (Caucasian, African American, Hispanic, or Asian), marital status (married, not married, or missing), employment status (full-time work, part-time work, retirement, or missing), number of children (0, 1–2, 3–4, 5 or more, or missing), and family history of diabetes (yes or no), smoking status (never smoked, past smoker, or currently smokes 1–14 cigarettes/d, currently smokes 15–24 cigarettes/d, or currently smokes ≥25 cigarettes/d, or missing), alcohol intake (gram/d: 0, 0.1–4.9, 5.0–14.9, or >15.0 in women; 0, 0.1–4.9, 5.0–29.9, or >30.0 in men; or missing), multivitamin use (yes, no, or missing), menopause status and postmenopausal hormones use (women only: premenopause, postmenopause [never, former, or current hormone use], or missing), physical activity (METs/week: 0–2.9, 3–8.9, 9–17.9, 18–26.9, ≥27.0, or missing), total energy intake (kcal/d), and baseline BMI (BMI not adjusted for obesity risk).

^b^ *p* < 0.01 for differences in weight changes compared to the reference group.

^c^ *p* < 0.001 for differences in weight changes compared to the reference group.

^d^ Study estimates from two cohorts were pooled using a fixed-effects model.

Further controlling for time-varying BMI attenuated the inverse association between MPAH and T2D (Table 4). We estimated that 57% (95% CI: 29%–81%; *p* < 0.001) of the association between MPAH and T2D was explained by BMI in women, and 24% (95% CI: 10%–48%; *p* < 0.001) was explained in men. The corresponding mediation effects were 62% (95% CI: 23%–90%; *p* < 0.001; women) for midday MPAH and 33% (95% CI: 19%–51%; *p* < 0.001; women) and 47% (95% CI: 10%–88%; *p* < 0.001; men) for evening MPAH. After further adjusting for dietary factors, the association between consuming MPAH and diabetes was attenuated when carbonated beverage intake was controlled for (Table 4). In women, carbonated beverage consumption was estimated to explain 22% (*p* < 0.001, for evening MPAH only) to 32% (*p* < 0.001, for both MPAH) of the association between MPAH and diabetes risk, whereas the values ranged from 23% (*p* < 0.001, for midday MPAH) to 41% (*p* < 0.001, for dinner) in men. Adjusting for fried food prepared away from home slightly attenuated the association for evening MPAH and overall MPAH. Other dietary factors did not explain the association between MPAH and T2D (Table 4 and S4 Table).

**Table 4:** Hazard ratios (95% confidence intervals) of type 2 diabetes according to frequencies of consuming meals prepared at home by further adjusting for BMI and dietary factors.

	Frequencies of Consuming MPAH, Times/Week				*P*-trend
**Overall Meals**	**0–6**	**7–8**	**9–10**	**11–14**	
+BMI^a^					
NHS	1.00	0.96 (0.88–1.05)	1.01 (0.93–1.11)	0.95 (0.88–1.03)	0.33
HPFS	1.00	0.92 (0.84–1.01)	0.96 (0.86–1.07)	0.94 (0.85–1.04)	0.22
Pooled^b^	1.00	0.94 (0.89–1.00)	0.99 (0.92–1.06)	0.95 (0.89–1.01)	0.13
*P*-heterogeneity	–	0.53	0.42	0.84	0.76
+Carbonated Beverage					
NHS	1.00	0.99 (0.91–1.08)	1.03 (0.94–1.13)	0.93 (0.85–1.01)	0.06
HPFS	1.00	0.92 (0.84–1.01)	0.98 (0.88–1.09)	0.95 (0.86–1.04)	0.30
Pooled	1.00	0.96 (0.90–1.02)	1.01 (0.94–1.08)	0.94 (0.88–1.00)	0.03
*P*-heterogeneity	–	0.23	0.45	0.78	0.68
+Fried Food away from Home					
NHS	1.00	0.99 (0.91–1.08)	1.01 (0.93–1.11)	0.89 (0.82–0.97)	0.003
HPFS	1.00	0.90 (0.83–0.99)	0.94 (0.85–1.05)	0.90 (0.82–0.99)	0.045
Pooled	1.00	0.95 (0.89–1.01)	0.98 (0.92–1.06)	0.90 (0.84–0.95)	<0.001
*P*-heterogeneity	–	0.15	0.33	0.89	0.73
+AHEI					
NHS	1.00	0.98 (0.90–1.06)	1.00 (0.91–1.09)	0.88 (0.81–0.95)	0.001
HPFS	1.00	0.88 (0.80–0.96)	0.92 (0.83–1.03)	0.85 (0.77–0.94)	0.002
Pooled	1.00	0.93 (0.88–0.99)	0.97 (0.90–1.04)	0.86 (0.81–0.92)	<0.001
*P*-heterogeneity	–	0.09	0.27	0.69	0.86
**Midday MPAH**	**0–2**	**3–4**	**5–7**		
+BMI					
NHS	1.00	1.00 (0.93–1.08)	1.00 (0.94–1.06)		0.92
HPFS	1.00	0.91 (0.82–1.00)	0.94 (0.86–1.02)		0.09
Pooled	1.00	0.96 (0.91–1.02)	0.98 (0.93–1.03)		0.29
*P*-heterogeneity	–	0.12	0.24		0.18
+Fried Food away from Home					
NHS	1.00	1.00 (0.93–1.08)	0.93 (0.88–0.99)		0.03
HPFS	1.00	0.93 (0.84–1.02)	0.91 (0.83–0.99)		0.02
Pooled	1.00	0.97 (0.92–1.04)	0.93 (0.88–0.97)		0.002
*P*-heterogeneity	–	0.22	0.61		0.54
+Carbonated Beverage					
NHS	1.00	1.02 (0.94–1.10)	0.97 (0.91–1.03)		0.28
HPFS	1.00	0.95 (0.86–1.05)	0.94 (0.86–1.02)		0.13
Pooled	1.00	0.99 (0.93–1.05)	0.96 (0.91–1.01)		0.08
*P*-heterogeneity	–	0.29	0.59		0.53
+AHEI					
NHS	1.00	1.00 (0.93–1.08)	0.93 (0.88–0.99)		0.03
HPFS	1.00	0.93 (0.84–1.02)	0.89 (0.81–0.97)		0.006
Pooled	1.00	0.97 (0.92–1.04)	0.92 (0.87–0.96)		0.001
*P*-heterogeneity	–	0.21	0.37		0.33
**Evening MPAH**	**0–2**	**3–4**	**5–7**		
+BMI					
NHS	1.00	0.99 (0.87–1.13)	0.91 (0.81–1.03)		0.01
HPFS	1.00	0.94 (0.80–1.10)	0.93 (0.80–1.08)		0.44
Pooled	1.00	0.97 (0.87–1.07)	0.92 (0.84–1.01)		0.02
*P*-heterogeneity	–	0.62	0.82		0.34
+Carbonated Beverage					
NHS	1.00	0.96 (0.84–1.10)	0.88 (0.78–0.99)		0.004
HPFS	1.00	0.92 (0.78–1.07)	0.92 (0.80–1.07)		0.55
Pooled	1.00	0.94 (0.85–1.04)	0.89 (0.82–0.99)		0.01
*P*-heterogeneity	–	0.66	0.63		0.18
+Fried Food away from Home					
NHS	1.00	0.96 (0.84–1.09)	0.87 (0.77–0.98)		0.002
HPFS	1.00	0.93 (0.80–1.09)	0.92 (0.79–1.07)		0.33
Pooled	1.00	0.95 (0.85–1.05)	0.89 (0.81–0.98)		0.002
*P*-heterogeneity	–	0.78	0.60		0.23
+AHEI					
NHS	1.00	0.95 (0.83–1.09)	0.84 (0.75–0.95)		<0.001
HPFS	1.00	0.92 (0.78–1.07)	0.87 (0.75–1.01)		0.06
Pooled	1.00	0.94 (0.85–1.04)	0.86 (0.78–0.94)		<0.001
*P*-heterogeneity	–	0.72	0.73		0.32

^a^ Estimates are hazard ratios (95% confidence intervals) from Cox proportional hazards models. All models were adjusted for age, ethnicity (Caucasian, African American, Hispanic, or Asian), marital status (married, not married, or missing), employment status (full-time work, part-time work, retirement, or missing), number of children (0, 1–2, 3–4, 5 or more, or missing), and family history of diabetes (yes or no), smoking status (never smoked, past smoker, or currently smokes 1–14 cigarettes/d, currently smokes 15–24 cigarettes/d, or currently smokes ≥25 cigarettes/d, or missing), alcohol intake (gram/day: 0, 0.1–4.9, 5.0–14.9, or >15.0 in women; 0, 0.1–4.9, 5.0–29.9, or >30.0 in men; or missing), multivitamin use (yes, no, or missing), menopause status and postmenopausal hormones use (women only: premenopause, postmenopause [never, former, or current hormone use], or missing], physical activity (METs/week: 0–2.9, 3–8.9, 9–17.9, 18–26.9, ≥27.0, or missing), and total energy intake (kcal/d) in addition to the covariable of interest.

^b^ Study estimates from two cohorts were pooled using a fixed-effects model.

We did not observe significant interactions of MPAH with age, BMI, physical activity, or smoking status on T2D risk (S5 Table). In sensitivity analyses, the associations between MPAH and T2D remained after participants who retired or divorced/separated were censored or when patients with incident T2D diagnosed in the first 4 y were excluded (S6 Table). In the NHS, further adjusting for the highest degree attained, the education attainment of the participant’s husband, the participant’s perception on her standing in US society, or the participant’s perception on her standing in the local community did not materially change the association between MPAH and T2D (S7 Table).

## Discussion

In two large prospective cohort studies of US men and women, we found that more frequent consumption of MPAH was inversely associated with T2D risk during 24–26 y of follow-up. The association between eating MPAH and T2D risk was independent of established and potential demographic, socioeconomic, lifestyle, and dietary risk factors of diabetes and was partially explained by less weight gain among participants who consumed MPAH more frequently. When analyzed separately, both midday and evening MPAH frequencies were associated with lower risks of obesity and T2D, and the associations for evening MPAH tended to be stronger than those for midday MPAH. Our study is among the first to explicitly examine MPAH in relation to T2D risk, and thus enables a more comprehensive understanding regarding the health impact of the current trend in changing from eating homemade meals to dining out. These findings provide further evidence to support reducing eating out frequency and increasing consumption of homemade meals for the prevention of excess weight gain and diabetes [29].

Because having more midday and evening MPAH is generally accompanied by less consumption of MPOH, our findings are in line with previous studies that linked meals cooked away from home, especially fast foods, with an increased risk of diabetes. For example, fast-food consumption has been associated with a higher risk of gestational diabetes among 3,048 Spanish women [13]. In the Coronary Artery Risk Development in Young Adults (CARDIA) study, consuming fast foods was associated with increased insulin resistance after 10–13 y of follow-up [15]. However, dining out in sit-down restaurants or cafeterias was not associated with insulin resistance [30]. Higher consumption of fried chicken, fried fish, and Chinese food in sit-down and fast-food restaurants was positively associated with a 10-y diabetes risk in the Black Women’s Health Study (BWHS), whereas eating fried chicken and fried fish prepared at home was not [14]. In the NHS and HPFS, consumption of fried foods cooked away from home, but not those made at home, has also been associated with a higher risk of T2D and coronary heart disease [16]. Fried foods prepared away from home and carbonated beverages are often consumed at fast-food restaurants [16,31], and adjusting for these foods/beverages partly attenuated the associations between MPAH and T2D in the current study. Therefore, participants eating more MPAH may benefit from lower fast-food consumption but also from consuming MPAH as a healthy dining option per se.

We also found that participants consuming more MPAH had less weight gain and a lower risk of developing obesity. Similarly, previous studies focusing on MPOH demonstrated that frequent consumption of foods in sit-down and fast-food restaurants was associated with excess weight gain as a result of prolonged positive energy balance [14,15,30–34]. For instance, each serving/week of fast-food consumption at baseline was associated with 0.32–0.74 kg more weight gain over 15 y in the CARDIA study [15,30,32]. More importantly, we found that BMI during follow-up partly explained the association between MPAH and T2D. In BWHS, consuming more MPOH was associated with a higher risk of developing obesity among initially lean participants [31], and adjusting for baseline BMI markedly attenuated the association between MPOH and T2D risk [14]. Clearly, future studies are needed to explicitly investigate the association of consuming MPAH/MPOH with other obesity-related diseases and potential intermediate role of weight gain.

Interestingly, both midday and evening MPAH were associated with risks of obesity and T2D in our study, and the associations were stronger for evening MPAH than for midday MPAH. One possible reason is the higher contribution of evening meals to total energy and nutrient intake for Americans [35]. Over the past four decades, daily energy from lunch was typically between 23.3% and 26.8% among US adults, and the range for dinner was 36.2%–38.8% [35], which may lead to a greater health impact of dinner than lunch.

The strengths of our investigation include large sample sizes, long follow-up durations, and repeated measurements of lifestyle and dietary factors in the analysis. However, our research is subject to a few limitations. First, although our questions regarding MPAH explicitly inquired about the location of meal preparation instead of consumption, we cannot further distinguish the processing extent or nutritional profile of ingredients used for MPAH. Homemade meals could be based on either convenience foods or raw/fresh ingredients, and evidence suggests that ready-to-eat convenience foods purchased from grocery stores and supermarkets have poorer nutritional profiles than raw, unprocessed foods [36]. If these foods were considered as MPAH, the expected association between MPAH and T2D could have been weakened. However, it has been shown that older participants consider that preparing meals from scratch is an essential component of cooking more than young people do [37]. Based on this evidence, in our middle-aged and older NHS and HPFS participants, MPAH is more likely to be based on raw, unprocessed materials. Varying but unclear definitions of MPAH/MPOH in existing literature have been a major challenge for interpreting related findings [35]. In this regard, future studies need to carefully assess locations of meal preparation/consumption, ingredients/foods used on each occasion, serving size and eating frequency, extent of food preparation/cooking, and these factors’ interrelationships to facilitate further understanding of how MPAH/MPOH influences human health [5,12]. Second, our participants are exclusively health professionals and mainly Caucasians, limiting generalizability of our findings to populations with similar ethnic and socioeconomic characteristics. On the other hand, the homogeneous socioeconomic status of our participants also reduces the residual confounding by socioeconomic factors. Third, measurement errors in self-reported assessments are inevitable. However, validation studies in the two cohorts demonstrated reasonable validity of self-reported assessments of diet, body weight, and diabetes incidence. For example, the correlation coefficient between self-reported and measured body weight was 0.97 [36]. Fourth, we cannot exclude the role of residual or unmeasured confounding. The adjustment of employment status, marriage status, and number of children may not fully account for differences in socioeconomic status. Fifth, we observed higher energy intake among participants with more MPAH. It is likely that participants who prepared their own meals may be more conscious about the food that they ate compared with those who consumed their food more passively, leading to underestimated food and total energy intake by participants with fewer MPAH. Therefore, our investigation was not able to determine the role of total energy intake in the associations of MPAH consumption and the risks of obesity and T2D, and findings regarding food intakes across MPAH groups need to be interpreted with caution. Lastly, because of the observational design of the two cohorts, we were unable to establish a causal relationship between consuming MPAH and T2D. Our findings warrant replication in intervention settings as well as observational studies in other populations with various demographic and socioeconomic characteristics.

In conclusion, frequent consumption of MPAH is inversely associated with T2D risk, which is partly attributed to less weight gain among individuals who consumed more MPAH. From a public health prospective, it is critical to facilitate people’s access to healthful, affordable foods and cultivate their confidence and skills for food preparation to encourage the practice of cooking meals at home [17]. Meanwhile, as needs for quick and convenient meals remain [9,37], actions are needed to improve the diet quality of MPOH, including fast-food chain restaurants, full-service restaurants, convenience stores, vending machines, and workplace and school cafeterias, so that the public’s need for convenience meals can be met while the diet quality of Americans is improved.

## Supporting Information

S1 Fig Flow diagram of study screening. (TIF)

S1 Table Baseline characteristics in the NHS and the HPFS according to frequencies of consuming midday MPAH. (DOCX)

S2 Table Baseline characteristics in the NHS and the HPFS according to frequencies of consuming evening MPAH. (DOCX)

S3 Table HRs (95% CIs) of T2D according to overall MPAH at baseline (DOCX)

S4 Table Pooled HRs (95% CIs) of T2D according to frequencies of midday and evening MPAH by further adjusting for dietary factors. (DOCX)

S5 Table Stratified analysis of HRs (95% CIs) of T2D according to frequencies of consuming MPAH. (DOCX)

S6 Table Sensitivity analysis on associations of MPAH and T2D risk. (DOCX)

S7 Table Sensitivity analysis on associations between frequencies of MPAH and T2D risk by further adjusting for other socioeconomic variables available in the NHS. (DOCX)

S1 Text STROBE checklist. (DOCX)

S2 Text Study proposal. (DOCX)

## Abbreviations

2010AHEI: 2010 Alternative Health Eating Index
BMI: body mass index
BWHS: Black Women’s Health Study
CARDIA: Coronary Artery Risk Development in Young Adults
CI: confidence interval
FFQ: food frequency questionnaire
HPFS: Health Professionals Follow-up Study
HR: hazard ratio
MET: metabolic equivalent task
MPAH: meals prepared at home
MPOH: meals prepared out of home
NHS: Nurses’ Health Study
T2D: type 2 diabetes
