# Supplementary material for: Consumption of Meals Prepared at Home and Risk of Type 2 Diabetes: An Analysis of Two Prospective Cohort Studies
Source: PLoS Med. 2016 Jul 5;13(7):e1002052. doi: 10.1371/journal.pmed.1002052 (PMC4933392; doi:10.1371/journal.pmed.1002052)
Supplement: S1 Table — (DOCX) [file pmed.1002052.s002.docx]

**S1 Table** Baseline characteristics in the NHS and the HPFS according to frequencies of consuming midday MPAH.

|  | **Frequencies of having meals prepared at home, times/week** | | | | | |
| --- | --- | --- | --- | --- | --- | --- |
| **Variables** ^a^ | **Nurse’s Health Study** | | | **Health Professionals’ Follow-up Study** | | |
|  | **0~2** | **3~4** | **5~7** | **0~2** | **3~4** | **5~7** |
| Number of participants | 26345 | 8990 | 22716 | 23608 | 6444 | 11624 |
| Number of evening meals prepared at home | 5.2(1.5) | 5.4(1.2) | 5.7(1.0) | 4.8(1.6) | 5.2(1.2) | 5.7(0.8) |
| Age, years | 50.6(6.5) | 51.5(7.2) | 54.1(7.3) | 50.9(8.5) | 53.9(9.9) | 56.8(9.9) |
| Race, white, % | 97 | 98 | 99 | 94 | 95 | 96 |
| Married, % | 78 | 85 | 87 | 89 | 92 | 94 |
| Number of children | 2.7(1.6) | 2.9(1.6) | 3.0(1.8) | 2.7(1.6) | 2.8(1.6) | 2.9(1.7) |
| Current working, % | 82 | 70 | 56 | 90 | 79 | 78 |
| Family history of diabetes, % | 26 | 25 | 26 | 19 | 19 | 19 |
| Physical activity, METs/week | 13.0(19.0) | 14.8(19.7) | 15.2(21.4) | 21.4(29.5) | 21.5(30.0) | 20.8(29.8) |
| Current smoking, % | 24 | 18 | 18 | 10 | 9 | 9 |
| Alcohol intake, g/day | 6.5(10.8) | 6.4(10.3) | 5.9(10.5) | 11.4(15.2) | 11.1(15.0) | 11.3(16.0) |
| Multivitamin use,% | 41 | 43 | 43 | 42 | 44 | 40 |
| Any use of postmenopausal hormone, % | 26 | 27 | 26 | - | - | - |
| Body mass index, kg/m^2^ | 25.2(4.6) | 25.2(4.6) | 24.9(4.6) | 24.9(5.0) | 25.0(4.8) | 24.8(4.7) |
| Dietary variables |  |  |  |  |  |  |
| Total energy, kcal/day | 1701(522) | 1810(528) | 1843(521) | 1937(618) | 2041(629) | 2091(610) |
| Total fruits, serving/day | 2.4(1.5) | 2.6(1.5) | 2.6(1.5) | 2.3(1.6) | 2.4(1.6) | 2.4(1.6) |
| Total vegetables, serving/day | 3.0(1.6) | 3.2(1.6) | 3.3(1.6) | 3.0(1.7) | 3.1(1.7) | 3.1(1.7) |
| Red meats, serving/day | 1.0(0.6) | 1.1(0.7) | 1.1(0.7) | 1.1(0.8) | 1.2(0.8) | 1.2(0.9) |
| Processed meats, serving/day | 0.1(0.2) | 0.1(0.2) | 0.1(0.2) | 0.2(0.3) | 0.2(0.3) | 0.2(0.3) |
| Total dairy products, serving/day | 2.1(1.4) | 2.2(1.3) | 2.3(1.4) | 1.8(1.3) | 2.0(1.4) | 2.2(1.5) |
| Carbonated beverage, serving/day | 0.9(1.1) | 0.8(1.1) | 0.7(1.0) | 0.8(1.1) | 0.7(0.9) | 0.6(0.9) |
| Coffee consumption, cups/day | 2.6(1.8) | 2.4(1.7) | 2.3(1.8) | 4.4(2.9) | 4.1(2.9) | 3.8(3.0) |
| French fries, serving/day | 0.1(0.1) | 0.1(0.1) | 0.1(0.1) | 0.1(0.2) | 0.1(0.2) | 0.1(0.1) |
| Whole grains, g/day | 13.3(13.5) | 14.2(13.1) | 15.3(14.1) | 20.6(19.3) | 22.5(18.8) | 23.3(20.0) |
| Trans fatty acids, % energy | 1.7(0.5) | 1.7(0.5) | 1.7(0.5) | 1.3(0.5) | 1.3(0.5) | 1.3(0.5) |
| P/S ratio | 0.5(0.2) | 0.6(0.2) | 0.6(0.2) | 0.6(0.2) | 0.6(0.2) | 0.6(0.2) |
| Sodium, g/day | 2.9(1.1) | 2.9(1.0) | 2.8(1.0) | 3.2(1.1) | 3.3(1.1) | 3.3(1.1) |
| AHEI ^b^ | 45.3(10.2) | 45.8(10.2) | 45.9(10.6) | 46.8(10.7) | 46.7(10.7) | 46.3(11.1) |
| Frequency of eating fried food away from home | 0.8(0.7) | 0.8(0.6) | 0.7(0.5) | 1.5(1.3) | 1.3(1.1) | 0.9(0.7) |
| Frequency of eating fried food at home | 1.1(1.0) | 1.1(1.0) | 1.2(1.2) | 1.3(1.2) | 1.5(1.3) | 1.6(1.5) |

^a^ Values are means (standard deviations) or percentages standardized to the age distribution of the study population.

^b^ Alcohol consumption was not included in the 2010 Alternative Health Eating Index score.
