# Supplementary material for: Consumption of Meals Prepared at Home and Risk of Type 2 Diabetes: An Analysis of Two Prospective Cohort Studies
Source: PLoS Med. 2016 Jul 5;13(7):e1002052. doi: 10.1371/journal.pmed.1002052 (PMC4933392; doi:10.1371/journal.pmed.1002052)
Supplement: S2 Table — (DOCX) [file pmed.1002052.s003.docx]

**S2 Table** Baseline characteristics in the NHS and the HPFS according to frequencies of consuming evening MPAH.

|  | **Frequencies of consuming evening MPAH, times/week** | | | | | |
| --- | --- | --- | --- | --- | --- | --- |
| **Variables** ^a^ | **NHS** | | | **HPFS** | | |
|  | **0~2** | **3~4** | **5~7** | **0~2** | **3~4** | **5~7** |
| Number of participants | 2303 | 8404 | 47344 | 2105 | 9991 | 29580 |
| Number of midday meals prepared at home | 2.4(2.1) | 2.5(2.0) | 3.5(2.3) | 1.4(1.3) | 2.0(1.7) | 3.1(2.3) |
| Age, years | 52.0(6.5) | 51.4(7.0) | 52.2(7.2) | 52.2(8.9) | 52.2(9.3) | 53.3(9.6) |
| Race, white, % | 97 | 98 | 98 | 95 | 95 | 95 |
| Married, % | 61 | 76 | 85 | 68 | 87 | 94 |
| Number of children | 2.7(1.7) | 2.6(1.6) | 2.9(1.7) | 2.4(1.7) | 2.6(1.6) | 2.9(1.6) |
| Current working, % | 81 | 75 | 68 | 91 | 90 | 89 |
| Family history of diabetes, % | 27 | 26 | 26 | 19 | 20 | 19 |
| Physical activity, METs/week | 13.7(23.4) | 14.2(19.0) | 14.2(20.3) | 21.0(29.4) | 23.0(30.6) | 20.9(29.6) |
| Current smoking, % | 28 | 21 | 21 | 12 | 9 | 10 |
| Alcohol intake, g/day | 5.7(11.2) | 6.4(10.5) | 6.3(10.7) | 10.6(15.1) | 11.1(14.8) | 11.5(15.6) |
| Multivitamin use,% | 47 | 45 | 42 | 46 | 44 | 41 |
| Any use of postmenopausal hormone, % | 28 | 28 | 26 | - | - | - |
| Body mass index, kg/m^2^ | 25.5(4.9) | 25.2(4.7) | 25.1(4.6) | 25.0(5.1) | 25.0(5.1) | 24.9(4.8) |
| Dietary variables |  |  |  |  |  |  |
| Total energy, kcal/day | 1648(560) | 1715(536) | 1790(520) | 1895(634) | 1945(632) | 2021(615) |
| Total fruits, serving/day | 2.2(1.5) | 2.4(1.5) | 2.5(1.5) | 2.1(1.7) | 2.3(1.6) | 2.4(1.6) |
| Total vegetables, serving/day | 2.7(1.7) | 3.0(1.6) | 3.2(1.6) | 2.7(1.7) | 2.9(1.7) | 3.1(1.7) |
| Red meats, serving/day | 0.9(0.7) | 1.0(0.6) | 1.1(0.7) | 1.0(0.8) | 1.1(0.8) | 1.2(0.8) |
| Processed meats, serving/day | 0.1(0.2) | 0.1(0.2) | 0.1(0.2) | 0.2(0.3) | 0.2(0.3) | 0.2(0.3) |
| Total dairy products, serving/day | 2.1(1.5) | 2.1(1.4) | 2.2(1.4) | 1.7(1.4) | 1.8(1.3) | 2.0(1.4) |
| Carbonated beverage, serving/day | 1.0(1.3) | 0.9(1.2) | 0.8(1.0) | 1.0(1.2) | 0.9(1.1) | 0.7(1.0) |
| Coffee consumption, cups/day | 2.7(2.0) | 2.5(1.8) | 2.4(1.8) | 4.5(2.9) | 4.3(2.9) | 4.1(2.9) |
| French fries, serving/day | 0.1(0.1) | 0.1(0.1) | 0.1(0.1) | 0.1(0.2) | 0.1(0.2) | 0.1(0.1) |
| Whole grains, g/day | 14.2(15.4) | 14.2(13.8) | 14.2(13.5) | 20.0(21.6) | 20.5(18.3) | 22.1(19.5) |
| Trans fatty acids, % energy | 0.1(0.1) | 0.1(0.1) | 0.1(0.1) | 0.1(0.2) | 0.1(0.2) | 0.1(0.1) |
| P/S ratio | 0.5(0.2) | 0.5(0.2) | 0.6(0.2) | 0.6(0.2) | 0.6(0.2) | 0.6(0.2) |
| Sodium, g/day | 2.8(1.1) | 2.9(1.0) | 2.8(1.0) | 3.2(1.2) | 3.2(1.1) | 3.3(1.1) |
| AHEI ^b^ | 45.1(10.7) | 45.9(10.2) | 45.6(10.4) | 46.2(10.8) | 47.1(10.7) | 46.5(10.8) |
| Frequency of eating fried food away from home | 1.2(1.1) | 1.0(0.8) | 0.7(0.5) | 2.0(1.7) | 1.6(1.3) | 1.1(1.0) |
| Frequency of eating fried food at home | 0.8(0.8) | 1.0(0.9) | 1.2(1.1) | 0.9(0.9) | 1.2(1.1) | 1.5(1.3) |

^a^ Values are means (standard deviations) or percentages standardized to the age distribution of the study population.

^b^ Alcohol consumption was not included in the 2010 Alternative Health Eating Index score.
