# Supplementary material for: Consumption of Meals Prepared at Home and Risk of Type 2 Diabetes: An Analysis of Two Prospective Cohort Studies
Source: PLoS Med. 2016 Jul 5;13(7):e1002052. doi: 10.1371/journal.pmed.1002052 (PMC4933392; doi:10.1371/journal.pmed.1002052)
Supplement: S3 Table — (DOCX) [file pmed.1002052.s004.docx]

**S3 Table** HRs (95%CIs) of T2D according to overall MPAH at baseline.

|  | | **Frequencies of consuming MPAH, times/week** | | | | **P**_trend_ |
| --- | --- | --- | --- | --- | --- | --- |
|  |  | 0-6 | 7-8 | 9-10 | 11-14 |  |
| NHS | Cases/ person-years | 1132/234306 | 1970/410027 | 891/190351 | 1961/447178 |  |
|  | Model 1^a^ | 1.00 | 1.00 (0.93, 1.07) | 0.97 (0.89, 1.06) | 0.90 (0.84, 0.97) | 0.004 |
|  | Model 2^b^ | 1.00 | 0.99 (0.92, 1.07) | 0.99 (0.91, 1.09) | 0.92 (0.85, 0.99) | 0.01 |
|  | Model 3^c^ | 1.00 | 0.97 (0.90, 1.05) | 0.98 (0.89, 1.07) | 0.87 (0.80, 0.94) | <0.001 |
| HPFS | Cases/ person-years | 1105/255971 | 1089/276962 | 440/111770 | 777/204749 |  |
|  | Model 1 | 1.00 | 0.91 (0.84, 1.00) | 0.90 (0.80, 1.01) | 0.85 (0.77, 0.94) | 0.001 |
|  | Model 2 | 1.00 | 0.92 (0.84, 1.00) | 0.90 (0.81, 1.01) | 0.85 (0.77, 0.94) | 0.001 |
|  | Model 3 | 1.00 | 0.92 (0.85, 1.00) | 0.91 (0.81, 1.02) | 0.85 (0.77, 0.93) | 0.001 |
| Pooled ^d^ | Model 2 | 1.00 | 0.96 (0.91, 1.02) | 0.96 (0.89, 1.03) | 0.89 (0.84, 0.95) | <0.001 |
|  | P for heterogeneity |  | 0.16 | 0.20 | 0.26 | 0.27 |
|  | Model 3 | 1.00 | 0.95 (0.90, 1.01) | 0.95 (0.88, 1.02) | 0.86 (0.81, 0.91) | <0.001 |
|  | P for heterogeneity |  | 0.67 | 0.52 | 0.76 | 0.68 |

^a^ Estimates are calculated in Cox proportional hazards models. Model 1, adjusted for age;

^b^ Model 2 was further adjusted for ethnicity (Caucasian, African American, Hispanic, or Asian), marital status (married, not married, or missing), employment status (full-time work, part-time work, retirement, or missing), number of children (0, 1–2, 3–4, 5 or more, or missing), and family history of diabetes (yes or no) based on model 1;

^c^ Model 3 was further adjusted for smoking status (never smoked, past smoker, or currently smokes 1–14 cigarettes/d, currently smokes 15–24 cigarettes/d, or currently smokes ≥25 cigarettes/d, or missing), alcohol intake (gram/d: 0, 0.1–4.9, 5.0–14.9, or >15.0 in women; 0, 0.1–4.9, 5.0–29.9, or >30.0 in men; or missing), multivitamin use (yes, no, or missing), menopause status and postmenopausal hormones use (women only: premenopause, postmenopause [never, former, or current hormone use], or missing), physical activity (METs/week: 0–2.9, 3–8.9, 9–17.9, 18–26.9, ≥27.0, or missing), and total energy intake (kcal/d) based on model 2;

^d^ Study estimates from the two cohorts were pooled using a fixed-effects model.
