# Supplementary material for: Consumption of Meals Prepared at Home and Risk of Type 2 Diabetes: An Analysis of Two Prospective Cohort Studies
Source: PLoS Med. 2016 Jul 5;13(7):e1002052. doi: 10.1371/journal.pmed.1002052 (PMC4933392; doi:10.1371/journal.pmed.1002052)
Supplement: S4 Table — (DOCX) [file pmed.1002052.s005.docx]

**S4 Table** Pooled HRs (95%CIs) of T2D according to frequencies of midday and evening MPAH by further adjusting for dietary factors ^a^

|  | **Frequencies of consuming MPAH, times/week** | | | | **P** _trend_ |
| --- | --- | --- | --- | --- | --- |
| **Both meals** | **0-6** | **7-8** | **9-10** | **11-14** |  |
| +Fried food at home |  |  |  |  |  |
| NHS | 1.00 | 0.97 (0.89, 1.05) | 0.99 (0.90, 1.08) | 0.86 (0.79, 0.93) | <0.001 |
| HPFS | 1.00 | 0.88 (0.80, 0.96) | 0.91 (0.81, 1.01) | 0.84 (0.76, 0.92) | 0.001 |
| Pooled | 1.00 | 0.93 (0.87, 0.98) | 0.95 (0.89, 1.02) | 0.85 (0.80, 0.91) | <0.001 |
| *P* _heterogeneity_ ^b^ |  | 0.10 | 0.24 | 0.70 | 0.86 |
| +Sodium |  |  |  |  |  |
| NHS | 1.00 | 0.97 (0.89, 1.05) | 0.99 (0.90, 1.08) | 0.86 (0.80, 0.94) | <0.001 |
| HPFS | 1.00 | 0.88 (0.80, 0.96) | 0.92 (0.82, 1.02) | 0.85 (0.77, 0.94) | 0.001 |
| Pooled | 1.00 | 0.93 (0.87, 0.99) | 0.96 (0.89, 1.03) | 0.86 (0.81, 0.91) | <0.001 |
| *P* _heterogeneity_ |  | 0.12 | 0.28 | 0.78 | 0.94 |
| +Fruit |  |  |  |  |  |
| NHS | 1.00 | 0.98 (0.90, 1.06) | 1.00 (0.91, 1.09) | 0.87 (0.80, 0.95) | <0.001 |
| HPFS | 1.00 | 0.89 (0.81, 0.97) | 0.92 (0.83, 1.03) | 0.86 (0.78, 0.95) | 0.003 |
| Pooled | 1.00 | 0.93 (0.88, 0.99) | 0.97 (0.90, 1.04) | 0.87 (0.81, 0.92) | <0.001 |
| *P* _heterogeneity_ |  | 0.12 | 0.31 | 0.84 | 0.97 |
| +Vegetable |  |  |  |  |  |
| NHS | 1.00 | 0.97 (0.89, 1.05) | 0.99 (0.90, 1.08) | 0.86 (0.79, 0.93) | <0.001 |
| HPFS | 1.00 | 0.88 (0.81, 0.97) | 0.92 (0.83, 1.03) | 0.86 (0.78, 0.94) | 0.002 |
| Pooled | 1.00 | 0.93 (0.87, 0.99) | 0.96 (0.89, 1.03) | 0.86 (0.80, 0.91) | <0.001 |
| *P* _heterogeneity_ |  | 0.15 | 0.36 | 0.99 | 0.82 |
| +Red meat |  |  |  |  |  |
| NHS | 1.00 | 0.96 (0.88, 1.04) | 0.98 (0.90, 1.07) | 0.86 (0.79, 0.93) | <0.001 |
| HPFS | 1.00 | 0.86 (0.79, 0.95) | 0.90 (0.80, 1.00) | 0.83 (0.75, 0.91) | <0.001 |
| Pooled | 1.00 | 0.91 (0.86, 0.97) | 0.95 (0.88, 1.01) | 0.85 (0.79, 0.90) | <0.001 |
| *P* _heterogeneity_ |  | 0.10 | 0.21 | 0.55 | 0.66 |
| +Proceeded meat |  |  |  |  |  |
| NHS | 1.00 | 0.97 (0.89, 1.06) | 0.99 (0.90, 1.08) | 0.86 (0.79, 0.94) | <0.001 |
| HPFS | 1.00 | 0.87 (0.80, 0.95) | 0.91 (0.81, 1.01) | 0.84 (0.76, 0.93) | 0.001 |
| Pooled | 1.00 | 0.92 (0.87, 0.98) | 0.96 (0.89, 1.02) | 0.85 (0.80, 0.91) | <0.001 |
| *P* _heterogeneity_ |  | 0.08 | 0.24 | 0.72 | 0.89 |
| +Total dairy product |  |  |  |  |  |
| NHS | 1.00 | 0.98 (0.90, 1.06) | 0.99 (0.91, 1.09) | 0.87 (0.80, 0.94) | <0.001 |
| HPFS | 1.00 | 0.88 (0.81, 0.97) | 0.92 (0.83, 1.03) | 0.86 (0.78, 0.94) | 0.003 |
| Pooled | 1.00 | 0.93 (0.88, 0.99) | 0.96 (0.90, 1.03) | 0.86 (0.81, 0.92) | <0.001 |
| *P* _heterogeneity_ |  | 0.12 | 0.31 | 0.87 | 0.95 |
| +Coffee |  |  |  |  |  |
| NHS | 1.00 | 0.97 (0.89, 1.05) | 0.98 (0.89, 1.07) | 0.85 (0.78, 0.92) | <0.001 |
| HPFS | 1.00 | 0.88 (0.81, 0.97) | 0.92 (0.82, 1.03) | 0.85 (0.77, 0.94) | 0.002 |
| Pooled | 1.00 | 0.93 (0.87, 0.99) | 0.95 (0.89, 1.02) | 0.85 (0.80, 0.91) | <0.001 |
| *P* _heterogeneity_ |  | 0.14 | 0.41 | 0.91 | 0.71 |
| +French fries |  |  |  |  |  |
| NHS | 1.00 | 0.98 (0.90, 1.07) | 1.01 (0.92, 1.11) | 0.89 (0.82, 0.97) | 0.003 |
| HPFS | 1.00 | 0.90 (0.82, 0.99) | 0.94 (0.84, 1.05) | 0.90 (0.82, 1.00) | 0.05 |
| Pooled | 1.00 | 0.94 (0.89, 1.00) | 0.98 (0.92, 1.05) | 0.90 (0.84, 0.96) | <0.001 |
| *P* _heterogeneity_ |  | 0.16 | 0.34 | 0.83 | 0.70 |
| +Whole grain |  |  |  |  |  |
| NHS | 1.00 | 0.98 (0.90, 1.07) | 1.01 (0.92, 1.11) | 0.89 (0.82, 0.96) | 0.002 |
| HPFS | 1.00 | 0.90 (0.82, 0.98) | 0.95 (0.85, 1.06) | 0.89 (0.81, 0.98) | 0.027 |
| Pooled | 1.00 | 0.94 (0.89, 1.00) | 0.99 (0.92, 1.06) | 0.89 (0.83, 0.95) | <0.001 |
| *P* _heterogeneity_ |  | 0.16 | 0.39 | 0.95 | 0.78 |
| +P/S ratio |  |  |  |  |  |
| NHS | 1.00 | 0.98 (0.90, 1.06) | 1.00 (0.91, 1.09) | 0.87 (0.80, 0.95) | <0.001 |
| HPFS | 1.00 | 0.88 (0.80, 0.96) | 0.92 (0.82, 1.03) | 0.85 (0.77, 0.94) | 0.002 |
| Pooled | 1.00 | 0.93 (0.88, 0.99) | 0.97 (0.90, 1.04) | 0.86 (0.81, 0.92) | <0.001 |
| *P* _heterogeneity_ |  | 0.10 | 0.26 | 0.74 | 0.91 |
| +Trans fat |  |  |  |  |  |
| NHS | 1.00 | 0.97 (0.89, 1.06) | 0.99 (0.90, 1.08) | 0.87 (0.80, 0.94) | <0.001 |
| HPFS | 1.00 | 0.88 (0.81, 0.97) | 0.92 (0.83, 1.03) | 0.86 (0.78, 0.95) | 0.004 |
| Pooled | 1.00 | 0.93 (0.88, 0.99) | 0.96 (0.90, 1.03) | 0.86 (0.81, 0.92) | <0.001 |
| *P* _heterogeneity_ |  | 0.13 | 0.34 | 0.93 | 0.90 |
|  |  |  |  |  |  |
| **Lunch** | **0~2** | 3~4 | 5~7 |  |  |
| +Fried food at home |  |  |  |  |  |
| NHS | 1.00 | 1.00 (0.93, 1.08) | 0.92 (0.87, 0.98) |  | 0.01 |
| HPFS | 1.00 | 0.92 (0.83, 1.02) | 0.88 (0.81, 0.96) |  | 0.004 |
| Pooled | 1.00 | 0.97 (0.91, 1.03) | 0.91 (0.87, 0.96) |  | <0.001 |
| *P* _heterogeneity_ |  | 0.19 | 0.39 |  | 0.34 |
| +Sodium |  |  |  |  |  |
| NHS | 1.00 | 1.00 (0.93, 1.08) | 0.93 (0.87, 0.98) |  | 0.01 |
| HPFS | 1.00 | 0.92 (0.83, 1.02) | 0.88 (0.81, 0.97) |  | 0.004 |
| Pooled | 1.00 | 0.97 (0.91, 1.03) | 0.91 (0.87, 0.96) |  | <0.001 |
| *P* _heterogeneity_ |  | 0.19 | 0.40 |  | 0.35 |
| +Fruit |  |  |  |  |  |
| NHS | 1.00 | 1.00 (0.93, 1.08) | 0.93 (0.87, 0.98) |  | 0.02 |
| HPFS | 1.00 | 0.93 (0.84, 1.03) | 0.89 (0.82, 0.97) |  | 0.007 |
| Pooled | 1.00 | 0.97 (0.92, 1.03) | 0.91 (0.87, 0.96) |  | <0.001 |
| *P* _heterogeneity_ |  | 0.24 | 0.47 |  | 0.42 |
| +Vegetable |  |  |  |  |  |
| NHS | 1.00 | 1.00 (0.93, 1.08) | 0.92 (0.87, 0.98) |  | 0.01 |
| HPFS | 1.00 | 0.93 (0.84, 1.03) | 0.89 (0.82, 0.97) |  | 0.008 |
| Pooled | 1.00 | 0.97 (0.92, 1.03) | 0.91 (0.87, 0.96) |  | <0.001 |
| *P* _heterogeneity_ |  | 0.25 | 0.52 |  | 0.47 |
| +Red meat |  |  |  |  |  |
| NHS | 1.00 | 1.00 (0.93, 1.08) | 0.93 (0.87, 0.99) |  | 0.01 |
| HPFS | 1.00 | 0.91 (0.82, 1.01) | 0.87 (0.80, 0.95) |  | 0.002 |
| Pooled | 1.00 | 0.97 (0.91, 1.03) | 0.91 (0.87, 0.96) |  | <0.001 |
| *P* _heterogeneity_ |  | 0.15 | 0.26 |  | 0.22 |
| +Proceeded meat |  |  |  |  |  |
| NHS | 1.00 | 1.00 (0.92, 1.07) | 0.92 (0.87, 0.98) |  | 0.009 |
| HPFS | 1.00 | 0.92 (0.83, 1.01) | 0.88 (0.81, 0.96) |  | 0.004 |
| Pooled | 1.00 | 0.97 (0.91, 1.03) | 0.91 (0.86, 0.95) |  | <0.001 |
| *P* _heterogeneity_ |  | 0.19 | 0.44 |  | 0.38 |
| +Total dairy product |  |  |  |  |  |
| NHS | 1.00 | 1.00 (0.93, 1.08) | 0.93 (0.87, 0.98) |  | 0.014 |
| HPFS | 1.00 | 0.93 (0.84, 1.03) | 0.89 (0.82, 0.97) |  | 0.008 |
| Pooled | 1.00 | 0.97 (0.92, 1.03) | 0.91 (0.87, 0.96) |  | <0.001 |
| *P* _heterogeneity_ |  | 0.24 | 0.49 |  | 0.44 |
| +Coffee |  |  |  |  |  |
| NHS | 1.00 | 0.99 (0.91, 1.06) | 0.91 (0.85, 0.97) |  | 0.002 |
| HPFS | 1.00 | 0.92 (0.84, 1.02) | 0.89 (0.81, 0.97) |  | 0.007 |
| Pooled | 1.00 | 0.96 (0.91, 1.02) | 0.90 (0.86, 0.95) |  | <0.001 |
| *P* _heterogeneity_ |  | 0.31 | 0.69 |  | 0.63 |
| +French fries |  |  |  |  |  |
| NHS | 1.00 | 1.01 (0.93, 1.09) | 0.94 (0.89, 1.00) |  | 0.06 |
| HPFS | 1.00 | 0.93 (0.84, 1.03) | 0.91 (0.84, 1.00) |  | 0.03 |
| Pooled | 1.00 | 0.98 (0.92, 1.04) | 0.93 (0.89, 0.98) |  | 0.005 |
| *P* _heterogeneity_ |  | 0.21 | 0.57 |  | 0.50 |
| +Whole grain |  |  |  |  |  |
| NHS | 1.00 | 1.01 (0.93, 1.09) | 0.94 (0.89, 1.00) |  | 0.06 |
| HPFS | 1.00 | 0.95 (0.86, 1.05) | 0.91 (0.84, 1.00) |  | 0.04 |
| Pooled | 1.00 | 0.98 (0.93, 1.05) | 0.93 (0.89, 0.98) |  | 0.007 |
| *P* _heterogeneity_ |  | 0.33 | 0.57 |  | 0.53 |
| +P/S ratio |  |  |  |  |  |
| NHS | 1.00 | 1.00 (0.93, 1.08) | 0.93 (0.88, 0.99) |  | 0.03 |
| HPFS | 1.00 | 0.93 (0.84, 1.02) | 0.89 (0.82, 0.97) |  | 0.008 |
| Pooled | 1.00 | 0.98 (0.92, 1.04) | 0.92 (0.87, 0.97) |  | 0.001 |
| *P* _heterogeneity_ |  | 0.21 | 0.39 |  | 0.37 |
| +Trans fat |  |  |  |  |  |
| NHS | 1.00 | 1.00 (0.93, 1.08) | 0.93 (0.87, 0.98) |  | 0.02 |
| HPFS | 1.00 | 0.93 (0.84, 1.02) | 0.89 (0.82, 0.97) |  | 0.008 |
| Pooled | 1.00 | 0.97 (0.91, 1.03) | 0.92 (0.87, 0.96) |  | <0.001 |
| *P* _heterogeneity_ |  | 0.24 | 0.47 |  | 0.42 |
| **Dinner** | **0~2** | 3~4 | 5~7 |  |  |
| +Fried food at home |  |  |  |  |  |
| NHS | 1.00 | 0.94 (0.82, 1.08) | 0.84 (0.74, 0.95) |  | <0.001 |
| HPFS | 1.00 | 0.91 (0.78, 1.06) | 0.87 (0.75, 1.01) |  | 0.06 |
| Pooled | 1.00 | 0.93 (0.84, 1.03) | 0.85 (0.77, 0.93) |  | <0.001 |
| *P* _heterogeneity_ |  |  |  |  |  |
| +Sodium |  |  |  |  |  |
| NHS | 1.00 | 0.75 | 0.72 |  | 0.333 |
| HPFS | 1.00 | 0.95 (0.83, 1.09) | 0.85 (0.75, 0.96) |  | <0.001 |
| Pooled | 1.00 | 0.92 (0.78, 1.07) | 0.88 (0.76, 1.02) |  | 0.07 |
| *P* _heterogeneity_ |  | 0.94 (0.85, 1.04) | 0.86 (0.78, 0.94) |  | <0.001 |
| +Fruit |  | 0.74 | 0.71 |  | 0.32 |
| NHS | 1.00 |  |  |  |  |
| HPFS | 1.00 | 0.94 (0.82, 1.07) | 0.83 (0.74, 0.94) |  | <0.001 |
| Pooled | 1.00 | 0.91 (0.78, 1.06) | 0.87 (0.75, 1.01) |  | 0.06 |
| *P* _heterogeneity_ |  | 0.93 (0.84, 1.03) | 0.85 (0.77, 0.93) |  | <0.001 |
| +Vegetable |  | 0.76 | 0.66 |  | 0.26 |
| NHS | 1.00 |  |  |  |  |
| HPFS | 1.00 | 0.95 (0.83, 1.09) | 0.83 (0.74, 0.94) |  | <0.001 |
| Pooled | 1.00 | 0.90 (0.77, 1.05) | 0.85 (0.73, 0.98) |  | 0.01 |
| *P* _heterogeneity_ |  | 0.93 (0.84, 1.03) | 0.84 (0.76, 0.92) |  | <0.001 |
| +Red meat |  | 0.62 | 0.85 |  | 0.39 |
| NHS | 1.00 |  |  |  |  |
| HPFS | 1.00 | 0.95 (0.83, 1.09) | 0.85 (0.75, 0.96) |  | <0.001 |
| Pooled | 1.00 | 0.91 (0.78, 1.06) | 0.87 (0.75, 1.00) |  | 0.04 |
| *P* _heterogeneity_ |  | 0.93 (0.84, 1.03) | 0.85 (0.78, 0.94) |  | <0.001 |
| +Proceeded meat |  | 0.66 | 0.82 |  | 0.37 |
| NHS | 1.00 |  |  |  |  |
| HPFS | 1.00 | 0.95 (0.83, 1.08) | 0.84 (0.75, 0.95) |  | <0.001 |
| Pooled | 1.00 | 0.91 (0.78, 1.07) | 0.87 (0.75, 1.01) |  | 0.06 |
| *P* _heterogeneity_ |  | 0.93 (0.84, 1.03) | 0.85 (0.78, 0.94) |  | <0.001 |
| +Total dairy product |  | 0.73 | 0.72 |  | 0.31 |
| NHS | 1.00 |  |  |  |  |
| HPFS | 1.00 | 0.94 (0.82, 1.08) | 0.83 (0.74, 0.94) |  | <0.001 |
| Pooled | 1.00 | 0.91 (0.78, 1.06) | 0.87 (0.75, 1.01) |  | 0.06 |
| *P* _heterogeneity_ |  | 0.93 (0.84, 1.03) | 0.85 (0.77, 0.93) |  | <0.001 |
| +Coffee |  | 0.75 | 0.66 |  | 0.26 |
| NHS | 1.00 |  |  |  |  |
| HPFS | 1.00 |  |  |  |  |
| Pooled | 1.00 | 0.96 (0.84, 1.10) | 0.86 (0.76, 0.97) |  | 0.001 |
| *P* _heterogeneity_ |  | 0.92 (0.79, 1.08) | 0.91 (0.78, 1.06) |  | 0.31 |
| +French fries |  | 0.94 (0.85, 1.04) | 0.88 (0.80, 0.97) |  | 0.001 |
| NHS | 1.00 | 0.71 | 0.58 |  | 0.17 |
| HPFS | 1.00 |  |  |  |  |
| Pooled | 1.00 | 0.95 (0.83, 1.09) | 0.85 (0.75, 0.96) |  | <0.001 |
| *P* _heterogeneity_ |  | 0.92 (0.78, 1.07) | 0.89 (0.76, 1.03) |  | 0.11 |
| +Whole grain |  | 0.94 (0.84, 1.04) | 0.86 (0.78, 0.95) |  | <0.001 |
| NHS | 1.00 | 0.74 | 0.64 |  | 0.23 |
| HPFS | 1.00 |  |  |  |  |
| Pooled | 1.00 | 0.95 (0.83, 1.08) | 0.84 (0.75, 0.95) |  | <0.001 |
| *P* _heterogeneity_ |  | 0.91 (0.78, 1.06) | 0.87 (0.75, 1.01) |  | 0.05 |
| +P/S ratio |  | 0.93 (0.84, 1.03) | 0.85 (0.78, 0.94) |  | <0.001 |
| NHS | 1.00 | 0.69 | 0.76 |  | 0.33 |
| HPFS | 1.00 |  |  |  |  |
| Pooled | 1.00 | 0.95 (0.83, 1.08) | 0.84 (0.75, 0.95) |  | <0.001 |
| *P* _heterogeneity_ |  | 0.91 (0.78, 1.07) | 0.88 (0.76, 1.02) |  | 0.09 |
| +Trans fat |  | 0.93 (0.84, 1.03) | 0.86 (0.78, 0.94) |  | <0.001 |
| NHS | 1.00 | 0.72 | 0.67 |  | 0.25 |
| HPFS | 1.00 | 0.94 (0.82, 1.08) | 0.84 (0.74, 0.95) |  | <0.001 |
| Pooled | 1.00 | 0.91 (0.78, 1.06) | 0.87 (0.75, 1.01) |  | 0.06 |
| *P* _heterogeneity_ |  | 0.93 (0.84, 1.03) | 0.85 (0.77, 0.93) |  | <0.001 |

^a^ Estimates are calculated in Cox proportional hazards model after adjustment of age, ethnicity (Caucasian, African American, Hispanic, or Asian), marital status (married, not married, or missing), employment status (full-time work, part-time work, retirement, or missing), number of children (0, 1–2, 3–4, 5 or more, or missing), and family history of diabetes (yes or no), smoking status (never smoked, past smoker, or currently smokes 1–14 cigarettes/d, currently smokes 15–24 cigarettes/d, or currently smokes ≥25 cigarettes/d, or missing), alcohol intake (gram/d: 0, 0.1–4.9, 5.0–14.9, or >15.0 in women; 0, 0.1–4.9, 5.0–29.9, or >30.0 in men; or missing), multivitamin use (yes, no, or missing), menopause status and postmenopausal hormones use (women only: premenopause, postmenopause [never, former, or current hormone use], or missing), physical activity (METs/week: 0–2.9, 3–8.9, 9–17.9, 18–26.9, ≥27.0, or missing), and total energy intake (kcal/d); midday or evening meals prepared at home was mutually adjusted for each other;

^b^ Study estimates from the two cohorts were pooled using a fixed-effects model.
