# Supplementary material for: Consumption of Meals Prepared at Home and Risk of Type 2 Diabetes: An Analysis of Two Prospective Cohort Studies
Source: PLoS Med. 2016 Jul 5;13(7):e1002052. doi: 10.1371/journal.pmed.1002052 (PMC4933392; doi:10.1371/journal.pmed.1002052)
Supplement: S5 Table — (DOCX) [file pmed.1002052.s006.docx]

**S5 Table** Stratified analysis of HRs (95%CIs) of T2D according to frequencies of consuming MPAH ^a^

| **Variables** | | **Frequencies of consuming MPAH, times/week** | | | | ***P*** _trend_ | ***P*** _interaction_ | ***P*** _heterogeneity_ |
| --- | --- | --- | --- | --- | --- | --- | --- | --- |
| Overall MPAH | | 0-6 | 7-8 | 9-10 | *P* _heterogeneity_ |  |  |  |
| Age | <65 years | 1.00 | 0.93 (0.86, 1.01) | 0.94 (0.86, 1.04) | 0.86 (0.79, 0.94) | <0.001 | 0.49 | 0.56 |
|  | *P* _heterogeneity_ |  | 0.31 | 0.47 | 0.71 | 0.68 |  |  |
|  | ≥65 years | 1.00 | 0.94 (0.85, 1.03) | 0.98 (0.89, 1.09) | 0.87 (0.79, 0.96) | 0.001 |  |  |
|  | *P* _heterogeneity_ |  | 0.24 | 0.45 | 0.97 | 0.66 |  |  |
| Body mass index | <30 kg/m^2^ | 1.00 | 0.91 (0.84, 0.99) | 0.98 (0.89, 1.08) | 0.89 (0.81, 0.97) | 0.01 | 0.05 | 0.07 |
|  | *P* _heterogeneity_ |  | 0.08 | 0.21 | 0.29 | 0.41 |  |  |
|  | ≥30 kg/m^2^ | 1.00 | 0.97 (0.89, 1.06) | 0.96 (0.86, 1.06) | 0.93 (0.85, 1.03) | 0.17 |  |  |
|  | *P* _heterogeneity_ |  | 0.28 | 0.78 | 0.30 | 0.42 |  |  |
| Physical activity | <18 METs/week | 1.00 | 0.95 (0.88, 1.02) | 0.95 (0.87, 1.04) | 0.86 (0.79, 0.93) | <0.001 | 0.18 | 0.80 |
|  | *P* _heterogeneity_ |  | 0.70 | 0.30 | 0.78 | 0.79 |  |  |
|  | ≥18 METs/week | 1.00 | 0.92 (0.82, 1.03) | 0.99 (0.87, 1.12) | 0.88 (0.79, 0.99) | 0.02 |  |  |
|  | *P* _heterogeneity_ |  | 0.02 | 0.33 | 0.65 | 0.88 |  |  |
| Current smoking | No | 1.00 | 0.93 (0.87, 0.99) | 0.96 (0.89, 1.03) | 0.86 (0.80, 0.92) | <0.001 | 0.94 | 0.69 |
|  | *P* _heterogeneity_ |  | 0.11 | 0.49 | 0.96 | 0.72 |  |  |
|  | Yes | 1.00 | 0.97 (0.79, 1.19) | 1.04 (0.80, 1.34) | 0.86 (0.69, 1.07) | 0.23 |  |  |
|  | *P* _heterogeneity_ |  | 0.81 | 0.11 | 0.20 | 0.16 |  |  |
| Midday MPAH | | 0-2 | 3-4 | 5-7 |  |  |  |  |
| Age | <65 years | 1.00 | 0.98 (0.90, 1.07) | 0.92 (0.86, 0.99) |  | 0.03 | 0.90 | 0.53 |
|  | *P* _heterogeneity_ |  | 0.47 | 0.36 |  | 0.34 |  |  |
|  | ≥65 years | 1.00 | 0.97 (0.89, 1.06) | 0.91 (0.85, 0.97) |  | 0.01 |  |  |
|  | *P* _heterogeneity_ |  | 0.37 | 0.87 |  | 0.84 |  |  |
| Body mass index | <30 kg/m^2^ | 1.00 | 0.96 (0.88, 1.04) | 0.95 (0.89, 1.02) |  | 0.14 | 0.13 | 0.67 |
|  | *P* _heterogeneity_ |  | 0.33 | 0.78 |  | 0.70 |  |  |
|  | ≥30 kg/m^2^ | 1.00 | 0.96 (0.88, 1.05) | 0.95 (0.88, 1.03) |  | 0.19 |  |  |
|  | *P* _heterogeneity_ |  | 0.18 | 0.12 |  | 0.08 |  |  |
| Physical activity | <18 METs/week | 1.00 | 0.97 (0.90, 1.05) | 0.90 (0.85, 0.96) |  | 0.002 | 0.20 | 0.05 |
|  | *P* _heterogeneity_ |  | 0.26 | 0.23 |  | 0.21 |  |  |
|  | ≥18 METs/week | 1.00 | 0.99 (0.89, 1.11) | 0.92 (0.84, 1.01) |  | 0.08 |  |  |
|  | *P* _heterogeneity_ |  | 0.42 | 0.46 |  | 0.50 |  |  |
| Current smoking | No | 1.00 | 0.96 (0.90, 1.02) | 0.91 (0.86, 0.96) |  | <0.001 | 0.77 | 0.90 |
|  | *P* _heterogeneity_ |  | 0.35 | 0.52 |  | 0.48 |  |  |
|  | Yes | 1.00 | 1.17 (0.94, 1.46) | 0.91 (0.76, 1.10) |  | 0.48 |  |  |
|  | *P* _heterogeneity_ |  | 0.50 | 0.31 |  | 0.30 |  |  |
| Evening MPAH | | 0-2 | 3-4 | 5-7 |  |  |  |  |
| Age | <65 years | 1.00 | 0.96 (0.83, 1.10) | 0.84 (0.74, 0.95) |  | <0.001 | 0.07 | 0.38 |
|  | *P* _heterogeneity_ |  | 0.63 | 0.24 |  | 0.13 |  |  |
|  | ≥65 years | 1.00 | 0.90 (0.77, 1.04) | 0.87 (0.76, 1.00) |  | 0.07 |  |  |
|  | *P* _heterogeneity_ |  | 0.29 | 0.43 |  | 0.87 |  |  |
| Body mass index | <30 kg/m^2^ | 1.00 | 1.04 (0.89, 1.20) | 0.94 (0.82, 1.08) |  | 0.03 | 0.74 | 0.29 |
|  | *P* _heterogeneity_ |  | 0.97 | 0.97 |  | 0.98 |  |  |
|  | ≥30 kg/m^2^ | 1.00 | 0.92 (0.79, 1.06) | 0.88 (0.77, 1.01) |  | 0.07 |  |  |
|  | *P* _heterogeneity_ |  | 0.19 | 0.98 |  | 0.18 |  |  |
| Physical activity | <18 METs/week | 1.00 | 0.93 (0.82, 1.05) | 0.84 (0.75, 0.95) |  | <0.001 | 0.35 |  |
|  | *P* _heterogeneity_ |  | 0.90 | 0.58 |  | 0.29 | 0.45 |  |
|  | ≥18 METs/week | 1.00 | 0.91 (0.75, 1.10) | 0.86 (0.72, 1.03) |  | 0.08 |  |  |
|  | *P* _heterogeneity_ |  | 0.67 | 0.50 |  | 0.47 |  |  |
| Current smoking | No | 1.00 | 0.91 (0.81, 1.01) | 0.84 (0.76, 0.93) |  | <0.001 | 0.86 |  |
|  | *P* _heterogeneity_ |  | 0.89 | 0.53 |  | 0.21 |  | 0.36 |
|  | Yes | 1.00 | 1.30 (0.91, 1.84) | 0.98 (0.71, 1.35) |  | 0.12 |  |  |
|  | *P* _heterogeneity_ |  | 0.82 | 0.95 |  | 0.70 |  |  |

^a^ Estimates are calculated in Cox proportional hazards model after adjustment of age, ethnicity (Caucasian, African American, Hispanic, or Asian), marital status (married, not married, or missing), employment status (full-time work, part-time work, retirement, or missing), number of children (0, 1–2, 3–4, 5 or more, or missing), and family history of diabetes (yes or no), smoking status (never smoked, past smoker, or currently smokes 1–14 cigarettes/d, currently smokes 15–24 cigarettes/d, or currently smokes ≥25 cigarettes/d, or missing), alcohol intake (gram/d: 0, 0.1–4.9, 5.0–14.9, or >15.0 in women; 0, 0.1–4.9, 5.0–29.9, or >30.0 in men; or missing), multivitamin use (yes, no, or missing), menopause status and postmenopausal hormones use (women only: premenopause, postmenopause [never, former, or current hormone use], or missing), physical activity (METs/week: 0–2.9, 3–8.9, 9–17.9, 18–26.9, ≥27.0, or missing), and total energy intake (kcal/d), midday or evening meals prepared at home was mutually adjusted for each other; Study estimates from the two cohorts were pooled using a fixed-effects model.
