# Supplementary material for: Consumption of Meals Prepared at Home and Risk of Type 2 Diabetes: An Analysis of Two Prospective Cohort Studies
Source: PLoS Med. 2016 Jul 5;13(7):e1002052. doi: 10.1371/journal.pmed.1002052 (PMC4933392; doi:10.1371/journal.pmed.1002052)
Supplement: S6 Table — (DOCX) [file pmed.1002052.s007.docx]

**S6 Table** Sensitivity analysis on associations of MPAH and T2D risk

| **Variables** | **Frequencies of consuming MPAH, times/week** | | | | **P** _trend_ |
| --- | --- | --- | --- | --- | --- |
| **Overall MPAH** ^a^ | 0-6 | 7-8 | 9-10 | 11-14 |  |
| Model 1 ^b^ | 1.00 | 0.93 (0.86, 1.00) | 0.97 (0.89, 1.06) | 0.87 (0.81, 0.94) | <0.001 |
| *P* _heterogeneity_ |  | 0.06 | 0.31 | 0.53 | 0.71 |
| Model 2 ^c^ | 1.00 | 1.01 (0.94, 1.09) | 0.92 (0.87, 0.98) |  | 0.01 |
| *P* _heterogeneity_ |  | 0.80 | 0.82 |  | 0.80 |
| Model 3 ^d^ | 1.00 | 0.91 (0.80, 1.03) | 0.86 (0.76, 0.97) |  | 0.005 |
| *P* _heterogeneity_ |  | 0.29 | 0.60 |  | 0.79 |
| **Midday MPAH** | 0-2 | 3-4 | 5-7 |  |  |
| Model 1 ^b^ | 1.00 | 1.01 (0.94, 1.09) | 0.92 (0.87, 0.98) |  | 0.01 |
| *P* _heterogeneity_ |  | 0.80 | 0.82 |  | 0.80 |
| Model 2 ^c^ | 1.00 | 0.91 (0.80, 1.03) | 0.86 (0.76, 0.97) |  | 0.005 |
| *P* _heterogeneity_ |  | 0.29 | 0.60 |  | 0.79 |
| Model 3 ^d^ | 1.00 | 0.99 (0.90, 1.09) | 1.03 (0.92, 1.15) | 0.94 (0.85, 1.04) | 0.23 |
| *P* _heterogeneity_ |  | 0.17 | 0.91 | 0.77 | 0.58 |
| **Evening MPAH** | 0-2 | 3-4 | 5-7 |  |  |
| Model 1 ^b^ | 1.00 | 0.91 (0.80, 1.03) | 0.86 (0.76, 0.97) |  | 0.005 |
| *P* _heterogeneity_ |  | 0.29 | 0.60 |  | 0.79 |
| Model 2 ^c^ | 1.00 | 0.99 (0.90, 1.09) | 1.03 (0.92, 1.15) | 0.94 (0.85, 1.04) | 0.23 |
| *P* _heterogeneity_ |  | 0.17 | 0.91 | 0.77 | 0.58 |
| Model 3 ^d^ | 1.00 | 1.03 (0.94, 1.13) | 0.97 (0.90, 1.06) |  | 0.56 |
| *P* _heterogeneity_ |  | 0.83 | 0.69 |  | 0.70 |

^a^ Estimates are calculated in Cox proportional hazards model after adjustment of age, ethnicity (Caucasian, African American, Hispanic, or Asian), marital status (married, not married, or missing), employment status (full-time work, part-time work, retirement, or missing), number of children (0, 1–2, 3–4, 5 or more, or missing), and family history of diabetes (yes or no), smoking status (never smoked, past smoker, or currently smokes 1–14 cigarettes/d, currently smokes 15–24 cigarettes/d, or currently smokes ≥25 cigarettes/d, or missing), alcohol intake (gram/d: 0, 0.1–4.9, 5.0–14.9, or >15.0 in women; 0, 0.1–4.9, 5.0–29.9, or >30.0 in men; or missing), multivitamin use (yes, no, or missing), menopause status and postmenopausal hormones use (women only: premenopause, postmenopause [never, former, or current hormone use], or missing), physical activity (METs/week: 0–2.9, 3–8.9, 9–17.9, 18–26.9, ≥27.0, or missing), and total energy intake (kcal/d), midday or evening meals prepared at home was mutually adjusted for each other; Study estimates from the two cohorts were pooled using a fixed-effects model.

^b^, Participants fully retired after baseline censored;

^c^, Participants divorce/separated after baseline censored;

^d^, Participants reported incident T2D within 4 years since baseline was censored.
