# Supplementary material for: Consumption of Meals Prepared at Home and Risk of Type 2 Diabetes: An Analysis of Two Prospective Cohort Studies
Source: PLoS Med. 2016 Jul 5;13(7):e1002052. doi: 10.1371/journal.pmed.1002052 (PMC4933392; doi:10.1371/journal.pmed.1002052)
Supplement: S7 Table — (DOCX) [file pmed.1002052.s008.docx]

**S7 Table** Sensitivity analysis on associations between MPAH and T2D risk by further adjusting for other socioeconomic variables available in the NHS ^a^

|  | **Frequencies of consuming MPSH, times/week** | | | | **P** _trend_ |
| --- | --- | --- | --- | --- | --- |
| **Overall MPAH** | **0-6** | **7-8** | **9-10** | **11-14** |  |
| Model 1^b^ | 1.00 | 0.98 (0.90, 1.06) | 0.99 (0.91, 1.09) | 0.86 (0.80, 0.94) | 0.005 |
| Model 2^c^ | 1.00 | 0.97 (0.89, 1.06) | 1.00 (0.91, 1.09) | 0.87 (0.80, 0.94) | <0.005 |
| Model 3^d^ | 1.00 | 0.97 (0.90, 1.06) | 0.99 (0.91, 1.09) | 0.86 (0.79, 0.93) | <0.005 |
| Model 4^e^ | 1.00 | 0.97 (0.90, 1.06) | 0.99 (0.91, 1.09) | 0.86 (0.79, 0.94) | <0.005 |
| Model 5^f^ | 1.00 | 0.98 (0.90, 1.07) | 1.00 (0.92, 1.10) | 0.87 (0.81, 0.95) | 0.005 |
| **Midday MPAH** | **0-2** | **3-4** | **5-7** |  |  |
| Model 1^b^ | 1.00 | 1.00 (0.93, 1.08) | 0.92 (0.87, 0.98) |  | 0.006 |
| Model 2^c^ | 1.00 | 1.01 (0.94, 1.09) | 0.93 (0.88, 0.99) |  | 0.006 |
| Model 3^d^ | 1.00 | 1.00 (0.93, 1.08) | 0.92 (0.87, 0.98) |  | 0.006 |
| Model 4^e^ | 1.00 | 1.00 (0.93, 1.08) | 0.92 (0.87, 0.98) |  | 0.006 |
| Model 5^f^ | 1.00 | 1.01 (0.93, 1.09) | 0.93 (0.88, 0.99) |  | 0.006 |
| **Evening MPAH** | **0-2** | **3-4** | **5-7** |  |  |
| Model 1^b^ | 1.00 | 0.95 (0.83, 1.08) | 0.84 (0.75, 0.95) |  | 0.01 |
| Model 2^c^ | 1.00 | 0.96 (0.84, 1.09) | 0.85 (0.75, 0.96) |  | 0.01 |
| Model 3^d^ | 1.00 | 0.95 (0.83, 1.09) | 0.84 (0.75, 0.95) |  | 0.01 |
| Model 4^e^ | 1.00 | 0.95 (0.83, 1.09) | 0.84 (0.75, 0.95) |  | 0.01 |
| Model 5^f^ | 1.00 | 0.96 (0.84, 1.09) | 0.85 (0.75, 0.96) |  | 0.01 |

^a^ Estimates are calculated in Cox proportional hazards model after adjustment of age, ethnicity (Caucasian, African American, Hispanic, or Asian), marital status (married, not married, or missing), employment status (full-time work, part-time work, retirement, or missing), number of children (0, 1–2, 3–4, 5 or more, or missing), and family history of diabetes (yes or no), smoking status (never smoked, past smoker, or currently smokes 1–14 cigarettes/d, currently smokes 15–24 cigarettes/d, or currently smokes ≥25 cigarettes/d, or missing), alcohol intake (gram/d: 0, 0.1–4.9, 5.0–14.9, or >15.0 in women; 0, 0.1–4.9, 5.0–29.9, or >30.0 in men; or missing), multivitamin use (yes, no, or missing), menopause status and postmenopausal hormones use (women only: premenopause, postmenopause [never, former, or current hormone use], or missing), physical activity (METs/week: 0–2.9, 3–8.9, 9–17.9, 18–26.9, ≥27.0, or missing), and total energy intake (kcal/d), midday or evening meals prepared at home was mutually adjusted for each other; Study estimates from the two cohorts were pooled using a fixed-effects model;

^b^, further adjusted for highest degree acquired (registered nurse, bachelor degree, master degree and above, or missing);

^c^, further adjusted for education level of husband (some high school or below, high school graduate, college graduate, graduate school, or missing);

^d^, further adjusted for participants perception on their standing in US society (top 20%, 30%, 40%, 50%, <50%, or missing);

^e^, further adjusted for participants perception on their standing in local community (top 20%, 30%, 40%, 50%, <50%, or missing);

^f^, further adjusted for years of rotating night shift work (never, 1-2 years, 3-5 years, 6-14 years, 15 years and more, and missing).
