# Supplementary material for: Consumption of Meals Prepared at Home and Risk of Type 2 Diabetes: An Analysis of Two Prospective Cohort Studies
Source: PLoS Med. 2016 Jul 5;13(7):e1002052. doi: 10.1371/journal.pmed.1002052 (PMC4933392; doi:10.1371/journal.pmed.1002052)
Supplement: S1 Text — (DOCX) [file pmed.1002052.s009.docx]

STROBE Statement—checklist of items that should be included in reports of observational studies

|  | Item No. | Recommendation | paragraph numbers  (subheadings are not counted) | Relevant text from manuscript |
| --- | --- | --- | --- | --- |
| **Title and abstract** | 1 | (*a*) Indicate the study’s design with a commonly used term in the title or the abstract | Paragraph 1, Title  Paragraph 7, Abstract, Methods and Findings |  |
|  |  | (*b*) Provide in the abstract an informative and balanced summary of what was done and what was found | Paragraph 7, Abstract, Methods and Findings |  |
| Introduction | | | |  |
| Background/rationale | 2 | Explain the scientific background and rationale for the investigation being reported | Paragraph 9-11 |  |
| Objectives | 3 | State specific objectives, including any prespecified hypotheses | Paragraph 11 |  |
| Methods | | | |  |
| Study design | 4 | Present key elements of study design early in the paper | Paragraph 13 |  |
| Setting | 5 | Describe the setting, locations, and relevant dates, including periods of recruitment, exposure, follow-up, and data collection | Paragraph 13,14 |  |
| Participants | 6 | (*a*) *Cohort study*—Give the eligibility criteria, and the sources and methods of selection of participants. Describe methods of follow-up | Paragraph 13,14 |  |
|  |  | (*b*) *Cohort study*—For matched studies, give matching criteria and number of exposed and unexposed  *Case-control study*—For matched studies, give matching criteria and the number of controls per case | N.A. |  |
| Variables | 7 | Clearly define all outcomes, exposures, predictors, potential confounders, and effect modifiers. Give diagnostic criteria, if applicable | Paragraph 15-18 |  |
| Data sources/ measurement | 8* | For each variable of interest, give sources of data and details of methods of assessment (measurement). Describe comparability of assessment methods if there is more than one group | Paragraph 15-18 |  |
| Bias | 9 | Describe any efforts to address potential sources of bias | N.A. |  |
| Study size | 10 | Explain how the study size was arrived at | Paragraph 14, Figure S1 |  |

Continued on next page

| Quantitative variables | 11 | Explain how quantitative variables were handled in the analyses. If applicable, describe which groupings were chosen and why | Paragraph 16, 20 |  |
| --- | --- | --- | --- | --- |
| Statistical methods | 12 | (*a*) Describe all statistical methods, including those used to control for confounding | Paragraph 20-25 |  |
|  |  | (*b*) Describe any methods used to examine subgroups and interactions | Paragraph 24 |  |
|  |  | (*c*) Explain how missing data were addressed | Paragraph 20 |  |
|  |  | (*d*) *Cohort study*—If applicable, explain how loss to follow-up was addressed | Follow-up rate is high our study |  |
|  |  | (*e*) Describe any sensitivity analyses | Paragraph 24 |  |
| Results | | | | |
| Participants | 13* | (a) Report numbers of individuals at each stage of study—eg numbers potentially eligible, examined for eligibility, confirmed eligible, included in the study, completing follow-up, and analysed | Paragraph 14, Figure S1 |  |
|  |  | (b) Give reasons for non-participation at each stage | Paragraph 14, Figure S1 |  |
|  |  | (c) Consider use of a flow diagram | Figure S1 |  |
| Descriptive data | 14* | (a) Give characteristics of study participants (eg demographic, clinical, social) and information on exposures and potential confounders | Paragraph 26 |  |
|  |  | (b) Indicate number of participants with missing data for each variable of interest | Paragraph 20 |  |
|  |  | (c) *Cohort study*—Summarise follow-up time (eg, average and total amount) | Paragraph 28 |  |
| Outcome data | 15* | *Cohort study*—Report numbers of outcome events or summary measures over time | Paragraph 28 |  |
|  |  | *Case-control study—*Report numbers in each exposure category, or summary measures of exposure | N.A. |  |
|  |  | *Cross-sectional study—*Report numbers of outcome events or summary measures | N.A. |  |
| Main results | 16 | (*a*) Give unadjusted estimates and, if applicable, confounder-adjusted estimates and their precision (eg, 95% confidence interval). Make clear which confounders were adjusted for and why they were included | Table 2 |  |
|  |  | (*b*) Report category boundaries when continuous variables were categorized | For covariates, Paragraph 21. For exposure, all tables. |  |
|  |  | (*c*) If relevant, consider translating estimates of relative risk into absolute risk for a meaningful time period | Paragraph 28-30 |  |

Continued on next page

| Other analyses | 17 | Report other analyses done—eg analyses of subgroups and interactions, and sensitivity analyses | Paragraph 31 |  |
| --- | --- | --- | --- | --- |
| Discussion | | | | |
| Key results | 18 | Summarise key results with reference to study objectives | Paragraph 32 |  |
| Limitations | 19 | Discuss limitations of the study, taking into account sources of potential bias or imprecision. Discuss both direction and magnitude of any potential bias | Paragraph 35 |  |
| Interpretation | 20 | Give a cautious overall interpretation of results considering objectives, limitations, multiplicity of analyses, results from similar studies, and other relevant evidence | Paragraph 33, 36 |  |
| Generalisability | 21 | Discuss the generalisability (external validity) of the study results | Paragraph 35 |  |
| Other information | |  | | |
| Funding | 22 | Give the source of funding and the role of the funders for the present study and, if applicable, for the original study on which the present article is based | Contradict to journal requirement, provided at submission |  |

*Give information separately for cases and controls in case-control studies and, if applicable, for exposed and unexposed groups in cohort and cross-sectional studies.

**Note:** An Explanation and Elaboration article discusses each checklist item and gives methodological background and published examples of transparent reporting. The STROBE checklist is best used in conjunction with this article (freely available on the Web sites of PLoS Medicine at http://www.plosmedicine.org/, Annals of Internal Medicine at http://www.annals.org/, and Epidemiology at http://www.epidem.com/). Information on the STROBE Initiative is available at www.strobe-statement.org.
