# Supplementary material for: Consumption of Meals Prepared at Home and Risk of Type 2 Diabetes: An Analysis of Two Prospective Cohort Studies
Source: PLoS Med. 2016 Jul 5;13(7):e1002052. doi: 10.1371/journal.pmed.1002052 (PMC4933392; doi:10.1371/journal.pmed.1002052)
Supplement: S2 Text — (DOCX) [file pmed.1002052.s010.docx]

**Frequencies of meals prepared at home and risk of diabetes**

**Proposed authors:** Geng Zong (first), David Eisenberg, Frank B. Hu, and Qi Sun (senior)

**Proposal presentation:** Feb.02.2015, NHS and HPFS meeting at Channing Division of Network Medicine, Boston.

**Background:**

Existing studies have described a trend from having meals prepared at home (MPAH) to eating food away from home (FAFH) among Americans over the past 50 years ([1](#_ENREF_1)). According to NHANES 2005-2010, U.S. adults consumed 4 meals per week from FAFH on average, and over 50% of them had ≥3 meals/week ([2](#_ENREF_2)). FAFH has been shown to be associated with poor diet quality ([2](#_ENREF_2), [3](#_ENREF_3)) and higher body weight in both adolescents and adults ([2](#_ENREF_2), [4](#_ENREF_4), [5](#_ENREF_5)). A previous study from CARDIA reported that more than twice a week of fast food consumption at baseline was positively associated with weight gain, but not insulin resistance over 15 years of follow-up ([6](#_ENREF_6)). However, fewer studies have investigated associations of MPAH or FAFH and risk of type 2 diabetes.

**Objectives:**

- To evaluate the association between the frequency and meals prepared at home (MPAH) and risk of diabetes among U.S. men and women.
- Whether these associations were explained by BMI or overall diet quality, as measured by alternative Health Eating Index (aHEI).

**Study population:**

- The Nurses’ Health Study (NHS) 1986-2010.
- The Health Professionals Follow-up Study (HPFS) 1986-2010.

**Exclusions:**

- Baseline diabetes mellitus, CVD or cancer cases.
- Missing exposure information.
- Missing BMI or aHEI at baseline.

**Priori hypothesis:**

A higher frequency of eating MPAH is associated with a lower risk of developing type 2 diabetes.

**Exposure:**

In 1986, frequencies of lunch and dinner prepared at home were collected by the following questions:

- How often are your midday meals prepared at home?
- How often are your evening meals prepared at home?

Five possible answers were provided in NHS (never, 1-2/week, 3-4/week, 5-6/week, and 7 days), whereas four answers were provided in HPFS (never, 1-2/week, 3-4/week, and 5-7 days).

**Outcome:** risk of type 2 diabetes.

**Covariates:**

Age, ethnicity (white, non-white), marriage status (married, divorced, separated, widowed, or never married), living arrangement (living alone, with partner, with family, nursing home, or other living arrangement), family history of diabetes, smoking status (never smoked, past smoker, or current smoker), alcohol intake (g/d: 0,0.1-4.9, 5.0-14.9, and >15.0 in women, 0,0.1-4.9, 5.0-29.9, and >30.0 in men), postmenopausal status and menopausal hormone use (in NHS), multivitamin use, physical activity, total energy intake, BMI (kg/m^2^: <23, 23-24, 25-29.9, 30-34.9 and >35), and aHEI.

**Statistical analyses:**

- Participants in NHS responded as “5-6/week” and “7 days” will be combined for the consistency with HPFS. Median values for midday MPAH and evening MPAH will be used to generate the frequency of both meals prepared at home.
- BMI and aHEI will be adjusted separately based on a multivariate model to test their modifying effect on MPAH-diabetes associations.
- When potential modifying effect is suggested, associations of MPAH with weight change and aHEI will be further analyzed.

**Reference:**

1. Smith LP, Ng SW, Popkin BM. Trends in US home food preparation and consumption: analysis of national nutrition surveys and time use studies from 1965-1966 to 2007-2008. Nutr J 2013;12:45. doi: 10.1186/1475-2891-12-451475-2891-12-45 [pii].

2. Kant AK, Whitley MI, Graubard BI. Away from home meals: associations with biomarkers of chronic disease and dietary intake in American adults, NHANES 2005-2010. Int J Obes (Lond) 2014. doi: 10.1038/ijo.2014.183ijo2014183 [pii].

3. McGuire S. Todd J.E., Mancino L., Lin B-H. The impact of food away from home on adult diet quality. ERR-90, U.S. Department of Agriculture, Econ. Res. Serv., February 2010. Adv Nutr 2011;2(5):442-3. doi: 10.3945/an.111.000679000679 [pii].

4. Fulkerson JA, Farbakhsh K, Lytle L, Hearst MO, Dengel DR, Pasch KE, Kubik MY. Away-from-home family dinner sources and associations with weight status, body composition, and related biomarkers of chronic disease among adolescents and their parents. J Am Diet Assoc 2011;111(12):1892-7. doi: 10.1016/j.jada.2011.09.035S0002-8223(11)01657-9 [pii].

5. Ayala GX, Rogers M, Arredondo EM, Campbell NR, Baquero B, Duerksen SC, Elder JP. Away-from-home food intake and risk for obesity: examining the influence of context. Obesity (Silver Spring) 2008;16(5):1002-8. doi: 10.1038/oby.2008.34oby200834 [pii].

6. Pereira MA, Kartashov AI, Ebbeling CB, Van Horn L, Slattery ML, Jacobs DR, Jr., Ludwig DS. Fast-food habits, weight gain, and insulin resistance (the CARDIA study): 15-year prospective analysis. Lancet 2005;365(9453):36-42. doi: S0140-6736(04)17663-0 [pii]10.1016/S0140-6736(04)17663-0.
